# Detection of Spotted Fever Group *Rickettsia* DNA by Deep Sequencing

**DOI:** 10.3201/eid2311.170474

**Published:** 2017-11

**Authors:** Rikki M.A. Graham, Steven Donohue, Jamie McMahon, Amy V. Jennison

**Affiliations:** Queensland Department of Health, Coopers Plains, Queensland, Australia (R.M.A. Graham, J. McMahon, A.V. Jennison);; Townsville Public Health Unit, Townsville, Queensland, Australia (S. Donohue)

**Keywords:** Rickettsia, spotted fever group, genome, bacteria, DNA, sequence analysis, fever, etiology, rash, deep sequencing, Australia

## Abstract

After conventional molecular and serologic testing failed to diagnose the cause of illness, deep sequencing identified spotted fever group *Rickettsia* DNA in a patient’s blood sample. Sequences belonged to *R. honei*, the causative agent of Flinders Island spotted fever. Next-generation sequencing is proving to be a useful tool for clinical diagnostics.

When conventional laboratory tests cannot identify an etiologic agent, unbiased deep sequencing performed directly on a clinical sample has the potential to identify a probable cause of disease. We used deep sequencing to detect spotted fever group (SFG) *Rickettsia* DNA in the blood of a patient for whom diagnosis was not possible through conventional molecular and serologic testing.

In late 2016, a middle-aged woman was admitted to a regional hospital in Queensland, Australia, after 2 weeks of mild cough, myalgia, fever, and lethargy. The day before admission, she experienced a blanching rash and pains in her feet, after which her condition deteriorated and a definite petechial rash appeared. Chest radiographs showed atelectasis on 1 side. Meningococcal septicemia was suspected, and the patient was transferred to intensive care with septic shock. Despite treatment with inotropes and several antimicrobial drugs (including ceftriaxone, vancomycin, meropenem, doxycycline), the patient died the next morning.

Clinical testing did not identify an infectious disease agent in the patient’s blood; serologic test results for *Rickettsia* were negative. Because a limited amount of specimen remained for testing, we applied an unbiased deep-sequencing approach. We extracted DNA from the blood sample by using the MasterPure Complete DNA Purification Kit (Epicenter, Madison, WI, USA) and sequenced with the Ion Torrent PGM (Personal Genome Machine) workflow by using the Ion PGM IC 200 Kit and the Ion 316 Chip Kit, version 2 (Life Technologies, Carlsbad, CA, USA). A total of 3,627,903 sequences were generated and trimmed by using a minimum quality score of Q15 and minimum length of 50 bp. Of the reads generated, 251 matched bacterial DNA sequences (uploaded to GenBank as Bioproject PRJEB21107). The rest either matched human genome sequences and were filtered out (3,619,386 reads) or were unclassified (8,252 reads). 

We analyzed the reads for bacterial DNA by using 3 metagenomics tools: Kraken (*1*), PathoScope (*2*), and One Codex (https://www.onecodex.com). All 3 analyses returned similar results; ≈80% of classified reads (208/251 reads, 53,958 total nucleotides) matched sequences from SFG *Rickettsia* spp.; the remainder gave low-number, low-quality matches to other bacteria. Screening of reads for sequences matching 5 rickettsial genes (*rrs*, *ompA*, *ompB*, *gltA*, and *sca4*) found 1 read mapping to the *ompB* gene (Technical Appendix). This read was a 100% match (272/272 nt) to *R. honei ompB* (GenBank accession nos. AF123724.1, AF123711.1). The next highest match was to *R. parkerii ompB* (accession no. KY113111.1) at 99% (270/272 nt). We confirmed the presence of SFG *Rickettsia* DNA in the DNA extract of the sample by nested PCR and performed Sanger sequencing by using the Invitrogen SuperScript III One-Step RT-PCR system with primers (*3*) and in-house nested primers.

To narrow down the identification to species level, we further analyzed sequences matching *Rickettsia* spp. We downloaded all *Rickettsia* genomes available at the National Center for Biotechnology Information (https://www.ncbi.nlm.nih.gov/), complete and draft, and used them as reference sequences for mapping of the reads in CLC Genomics Workbench 8 (QIAGEN Aarhus, Silkeborgvej, Denmark). We discarded reads mapping to >1 genome, collected the remaining reads that mapped uniquely to a single genome, and noted the genome to which they mapped. Of the 208 reads, 67 mapped to >1 genome, 1 did not map to any of the genomes and was subsequently identified as matching that of the human reference genome, 3 were unique matches to *R. conorii* (AJUR01, GenBank accession no. NC_003103), 1 was a unique match to *R. sibirica* (accession no. NZ_AHZB01000018), and 151 were unique matches to *R. honei* (accession no. NZ_AJTT00000000) (Technical Appendix) (*4*). Mapping of the 208 sequencing reads revealed that 207 (99.6%) reads mapped to the *R. honei* genome, giving 1.43% coverage of the genome, and 168 (80.7%) reads mapped to the *R. australis* (accession no. NC_017058) genome, representing 0.03% coverage of the genome.

The main causes of SFG rickettsioses in Australia are *R. australis* and *R. honei*, which cause Queensland tick typhus and Flinders Island spotted fever, respectively (*5*). The rickettsial DNA in the blood sample we describe most closely matched sequences from *R. honei* and had a relatively low level of similarity to sequences from *R. australis*. *R. honei* was initially reported only in the southern states of Australia; however, a genetic variant known as the “marmionii” strain has since been reported in eastern and northern parts of the country (*6*). Unfortunately, the genome of *R. honei* “marmionii” has not been sequenced, and the genes used to differentiate between *R. honei* and *R. honei* “marmionii” were not covered by the sequences generated from the sample. Therefore, we could not confirm which strain of *R. honei* was in the sample.

Flinders Island spotted fever is reportedly associated with relatively mild illness (*5*). However, our detection of *R. honei* DNA in the blood of a deceased patient, in the absence of positive *Rickettsia* serologic test results, is suggestive of acute infection with this agent. This case demonstrates the potential of deep sequencing for identifying unknown etiologic agents, particularly when other methods have not done so.

Technical Appendix Characteristics of sequence reads mapping to *Rickettsia honei*.
