# Supplementary material for: Detection of Spotted Fever Group Rickettsia DNA by Deep Sequencing
Source: Emerg Infect Dis. 2017 Nov;23(11):1911–3. doi: 10.3201/eid2311.170474 (PMC5652451; doi:10.3201/eid2311.170474)
Supplement: Technical Appendix — Characteristics of sequence reads mapping to Rickettsia honei. [file 17-0474-Techapp-s1.pdf]

Article DOI: https://doi.org/10.3201/eid2311.170474

Detection of Spotted Fever Group *Rickettsia* DNA by Deep Sequencing

Technical Appendix

Technical Appendix Table. Characteristics of sequence reads mapping to *Rickettsia honei*

Read name	Sequence length	% ID	GenBank accession no.	nt coordinates	Sequence
700666F:214:CA2UUANXX:8:2211:4818:11819_1:N:0:TAGGCATG+CTCTCTAT_(reversed)	125	100	NZ_AJTT01000001	102663 to 102787	AGCTTTCAGTTTCAGTAATGATAGCTGTTTTACTTTGGAAATAAAGAGCTTTACCGATTAACTCAGTGCTTACTACATTAGTATCTCTTGCAAAAAATTCACGAGGTAAAGGGATTAGTTTATTC
LH08G:00089:01332	294	98.98	NZ_AJTT01000001	58385 to 58675	CACGCAGCAAGTCTTGATTTACCGGTTCCGGGAGGACCAAACATTAAAAGGTTATGCCATCTGATGCTGCAATTTCTAAAGCTTGTTTAGCAATTTTTTTGACCTTTTTATATCTTTAAAATCGGGATAATTTATCGCCTCATCTTGTAGCTTAGCTTCCGGAGGAGTTAAAAACTTGTGAACCTTTAAAATGATTTACTAATTCAATTAAATTACCTGCAACAAGTATATTATCATTACCCGACCAAGCAACTTCCGAGCTGTTTTTACTTGAACAAATCAGACCTTTACCTC
LH08G:00103:02050	405	96.79	NZ_AJTT01000001	235861 to 236252	TTAGCTGATTCTAACATAGCTTTTTCTAATGGTTTTTTAATATCCATCACTTCATCTTCGCTTAAGCCATTACGTTTTTTCGTCGTTTCACCTGCTCTACTTGAGACTCACTTAAATCTCTGGCAAACCAATTTAAAATTTTATGAATGAAAATTAATTAAAAACTTATAAAAAAGAATTGTATTTTTTTTCATAGAAGAGGTCCTGATCGTAATTTTCATCAGGGATTTTCAGTTATGAACCTTGAAGGATAAGAACGGACTATTTCATAAAATATTTTTTCTGCTTTCAGCATGAGTAATGTAGAGGTTCTTTTTTGGCTCTCGTAATACCGACATAAGCAATGCGGCGTTCTTCTTCTAAGCCTTTTTTCGCCGTCTTCGTCTAGAGATCTTTTGAGACGGA
LH08G:00199:02115	268	99.627	NZ_AJTT01000001	173179 to 173445	GAGGGTAGAGCGTCCAGCCCGTTCCTGGCCCACCGTCAACAAAAGCTGATCCCATAAGTAAAAGAAAAGCAGGCACTAGTAGCCAAAAACTGATATTGTTAAGGCGTGGAAATGCCATATCGGGAGCCCCTATTAACAGAGGTACGAAATAATTACCAAAGCCACTGAACAAAGCCGGCATAATCAATAAAGAATACCATAATAACCGCATGTGCTGTGATAAGCACGTTATATAGCTGGAAATCATGATTTAAGAAAGTGCCGCCGG
LH08G:00260:02605	310	99.032	NZ_AJTT01000001	304717 to 305023	CAAGTTTTAGACTAGGTATATTTAATATTGAATCATTAATCCATTTAATATTTTTAAGACCTCGTACTTCTGCACGTTTTTGTGCTACTTCCATGCTAGGCTTGCTGAAATCTAGATATATTATTTTCGGCATTTTTTATCTTTTAACTGCTCGGCAAGATAAATAGTCGAATCACCGGTGCCGCCGCCTGCTATTAAGCATCTAAAATTATTATTAAAATTTTCTTTTCCTTGATATAGAAAATGGTTTAATTCTCCTAAGAACTCACCGCATGTTGCAAGTAAATATTCTTTTTTTCTTGATTTGGGTC
LH08G:00384:01842	213	99.531	NZ_AJTT01000001	22382 to 22593	CTTTTTGTATCTCTATATTGTACAGTATCAAAATTGCTGCTACATCAAGCATAGATGTATCATCGATGTTATGTATATATCTATCGTATTCAGCAGAAGTAAAGTGGCTAGGGTTAGCATAAGAAATAATAAGTTTATATGCTTCTGGATAAAATGACGTAATGGCATGATGTAACCCTAACAAATTTATAGCTGTTCTTAGATGCTAAAATT

Read name	Sequence length	% ID	GenBank accession no.	nt coordinates	Sequence
LH08G:00577:01769	208	96.172	NZ_AJTT01000001	341663 to 341864	GTTATAAAAATAAATTTCGTCCATAGAGAAAGTCTAGCATAACAATGCATTAAAA GTAATTTAAATTTGTCAATATCAAGTTCATTGAAATTTATAAGCCTTTTGTTGCGT AATTTTCTGCTTTCTCATAAAAAGATTTATCATATAAAGTTTTTGCTAGATTTTTTG CTACCGTAAAAAACAAATTCTTTAGATGTAATAAGTTTTT
LH08G:00579:02051	362	100	NZ_AJTT01000001	124494 to 124855	GATACATAAATAATGCCATACTTTCAACAGGAGCATATTTAAGCCAATCATCAAT GCTAATACTATTACCTTTTGATTTTGATATTTTCTCACCATTTTCATCTAAGAATA ACTCATAACATAACTGCACCGGCGGCTTTCCTCCTAGGATTCGGCAAATTTCCG AATAAAGACGAGCATTTGCCAAATGGTCTTTGCCGTACATCTCATAATCGACTTT AAGCGCAGCCCAACGCATACCAAAATCCGGCTTCCACTGTAATTTGCAATGCCC TCCCGTTACCGGTACTTCTATATAATTACCGGCTTTGTCTTTATACGTTACAGTG CCTGCTTTAGCATCCCATTTCTCTATAGGTACT
LH08G:00581:02966	309	99.029	NZ_AJTT01000001	218033 to 218339	GTAGGAATTGCTGTTTTTGCTTATATTTTTATTATAATTAATGCAGAAACACTTTG GTGGCTTGCAGTTGGCTCATCCTTAATAATTATTGGAACATCTAATGATTCAGGA TTAAGTAAACTTATTTTCGGTAATCCAATAACATATTTTCTAGATCAAATTTCATA TGCTTTATACCTTTCTGCTTTAACTACAGAATTGTTTGTAGCATATTACTGGATTT TATCCAACTCCTTTATGGCTTAAATTTGCTACTTCGGTTACAATAGCTACTATTTT GCATTATATATAATTGAATTTACCAGCTCGCC
LH08G:00660:02650	139	97.744	NZ_AJTT01000001	347909 to 348039	ATGATAATATCTTGCAAATTTAGATATTTTAGGAGTATATTCCCCTAAATATCTAT TTATCTAGTTGATTTAAGAGGTTATCTGTAAATAAATTGTAATTAGCAAGTCAAGA TGCTTTTTTAGCTAAAATTGAATTAAG
LH08G:00737:02796	261	99.237	NZ_AJTT01000001	242578 to 242838	ACAATATTTTTTTGGTACAGCGTCGTTCAGAAAGCTTGATACAAGTGAATTTTCT GACATGCGCCTTTGCTCACGTACTTTTTGTACGTTCCGCAGGCTCAGTCGTCAA TTCATCTTGTCTAAAGCTTTCTGAATCTAGCTCTATCCACTTCTATTTTATTATTG TAAAAAAGTTGCTACAAATTATTAATTAAATGTATTAATATTAATAAAGAATCAAA AATATTAAGAAAATTTTTAGCAAACGGCATCATTATGCGG
LH08G:00813:00255	253	100	NZ_AJTT01000001	301855 to 302107	AGCAAAAATTAAAGAATATAATAAGGCTAAATCTGAGCTCGAAGAAGCAAATAAT TTTAAGCTAGAAGTGGGACTTGATAATGCCACTTTAGAAATGATTGAAGACGAA ATATACACTCTTGAAAATTCGTTACCAAAACTTGAAAGAGCCGTAAAAATTGCTT TATTACCAAAAGATGATGCGGATAGTAAAAGTGCTATTATTGAAGTTAGAGCAG GAAGCGGCGGAGAAGAAGCGGCACTTTTTGCTGCC
LH08G:00820:01524	258	100	NZ_AJTT01000001	81173 to 81430	ATTATCCATATCACTTTGGCTTAAGTAACTTTTACCGACTGCATAGGTAAATAGT GTAACATGCGGTCTTATAATAAAATGAATATTAGGATGTTGTAATGCATAATTAT AGAAAAAATAGTGATAATTTAAGAAAGTAGAACCTCCTTCATATAAATCTCTATTT TTAAATGATAATTCCCATCTAGGAAGCCATAAAATGGTTTCCGAGTTTGGTTTTT CTTTAATATTATAATTTCTTACTTCATAGTATGGTTG
LH08G:00832:00648	282	98.94	NZ_AJTT01000001	200820 to 201101	AAGATTTACCCGTTACTTTACCTGATGATGTAAATTTTGACGGTCACGGTAATCC GCTTGATCATCATCCTAGTTGGAAACATGTTAATTGTCCGAAATGCGATAAGCC GGCTGTGCGTGAAACCGATACTTTTGATACATTTTTTTGAATCACTTGGTATTTT ACGAGATATTGCAATAGCAATGTTACAGAGATGACTGATAAAAAAGCTTGTGATT ACTGGCTACCGGTCGATAAATATATAGGCGGCATTAAACATGCGGTAATGCATT TATTATATG
LH08G:00880:03124	260	100	NZ_AJTT01000001	302965 to 303224	AAAATTTCTTAAGACCGAAAATCAAAAATTTATTACCTGATCCTTTTCATCTACTT GATATGGAAAAAGCTGTAGATAGAACCGTTACAGCTATTTTAAATAACCAAAAAA TATGTATCTTCGCCGATTATGATGTAGACGGTGCTACTTCAGCCGCCTTACTTA AAAATATTTTTAGAGATTTAAATATACTATGTGACATTTATGTTCCTGATCGTATA GCTGAAGGATACGGTCCAACTCCTTTTGCTATGCAAAAA
LH08G:00901:00649	283	98.233	NZ_AJTT01000001	16343 to 16620	CTTATTAACCATAATTTCGTTAATACCGTCTTCAGCAAATAAATGTTTAAAAGGAA GTAAAAACGTCTCTAAGGCTGCAAATTCTTCATTCATTTCATTACCTTCGATTTTA

Read name	Sequence length	% ID	GenBank accession no.	nt coordinates	Sequence
					ATAAAACGGCTTTTGGGAATTTATAATCCTTATTAACATATATTCTAACCGGCGTACCTTGATTAACCGTTGTCGTCGGCTTAAACTGCTGCTGAGTTATCATATTTTTTTACAACATTCGTTTACATCCGTAAAAAGCTTGAGTAGAAGCTTTGTTGTGCTTTGCGTCGG
LH08G:00911:00432	271	98.893	NZ_AJTT01000001	354716 to 354983	AGTATTTAAACTTCTATAAATTTCGCTCAAAAATACAAGTAATAGATTGTAGTAACGAATATAAAGTTATCTACTCACTTCAAAAATTAGATATAGACTCGCTAATTACGGTTCGTGATCCTCGTTATGCTAAGCTTGGTTTCCGCTCAATAAATAAGCTCGATGTCATACCGCTGTGCGGTATCCAGACAATAATTAAAAAAAGACTGGACCCCGTGGTCAAGCCACAGGGTGACAGAGGGGGGAACCGCCCCCCCTCAACAACATCTCC
LH08G:00932:00966	290	98.966	NZ_AJTT01000001	370114 to 370400	CTATACCCCAAATGCTGAAAAAGACTGTTTTAATTCATCTTTCCCTATTATTCATACTAAAACAGATGCAAAACATAATACTTGGCTTCACATAGTTCGCACTGATTTGCTCGGATAAGGAATTACAAGAGTTTATAGACGGTGATATAAAACTCAATTACCCATTTTACACTTTAGAACAAGATTTTTTCTGATTCTCCTTTATGGTACTACACACTTTTTCTCTAAACCTTTATCTTACTGGATCGGTCACGTTTTATACCATTAAAATTGATCATGAGCGTAAAACA
LH08G:00996:01347	265	100	NZ_AJTT01000001	261795 to 262059	AAAATATTACCAGTTAGTATAGGAGCATTATTAAAAGGTGCGAAGTTTGCCTTCACTAATCCCGTAAATAGTTATAATCTTGTTAAACTTGCAAGCTCCTCTGATATATATCTTGATTCAAAGTTTCAAAAATCTGTTCTTGAAAAATCTGAAATATAGGATTTTCTAAAAAAGCATGCCGATGATTTACCAAAAATAGGACAAATCTTAGCAAAAGCAGGTTTTAGAGAATTTCAAGATAAGAGTGCTTTAGATGAAAAGGGAC
LH08G:01008:02633	232	99.138	NZ_AJTT01000001	82660 to 82889	CTAATTATTAGTGCAGAAGAAAAATCTATTTTTCTAAAGCTCTATAGAAAATTTAAAACAATAAAACTAACATTTTATAAATTATTTTTAAAAGTTATGACAACTAGAAAAGATAAACCTCTAAATATTTATCATACTTTAGTTTCCATTATTATACCTGTCTATAATGGGGCTAATTATATGAAAGAAGCTATAAATAGTGCTTTAGCACAAACTTTACAAAAATATTGAA
LH08G:01016:02876	294	98.639	NZ_AJTT01000001	273506 to 273795	TTGATTTTAGAGGAAGAATTTTCTATATTACTGGGGTTAATCGTCATTATTTGGTCTAACCTATCTTGCTATTGGCTAGAGTTATATATTAAAGATTGTCTAAATTTAAGTCAAGTTAAAAATCAAATTAAAATGAGTAATTATTGGTTAAATATTTATAAAACCAAGAGGTATAAGTTCTGCTCAACTTGTTAGCATAGTAAAAAAAAAATACTAGGTAAAACCAAGATAGGACATGCCGGTACTTTAGACGTAGAAGCGGAAGGAATATTACCGTTTGCCGTAGGTGAAGCT
LH08G:01054:02101	244	98.086	NZ_AJTT01000001	148127 to 148333	TTTCGTGTATTAGTGTTAACTTAATAGGAGAAAAAACCTCTTATGCCATTTCCGCGAAATCCGGAATCCAGTACTTTAAAGCTTTTTAAAAGCTCGATTTATCTTGCTTTACCCCTAGATTTCTGCTTTCGCAGGAATAACATCATATCACTGATTCAAAAAGAGATTAACATGAAAAATTTTATAAAATATTTAGTTTTTTACCTGGTTTTCACGTACTAATTAGTTAAGTGGTAACGGTGAA
LH08G:01072:01642	222	99.099	NZ_AJTT01000001	203673 to 203893	GGAGTAGCTGCTCCAAATGAGCAGGTGTTTAATCACCACTAACAAAACTTTGTTGTCATCCCGCGACTTGATCGCGGGATCCAGTCTTTTTTAATTTTTTTTTTGATACCGTGGTCAAGTCACGGTATGACACAGAACGCGTTTTTCGATCCGCACAACAACATTATTGATTCAAAGGAATTAAAAATCATTTATGACTATAAAAATTTATCAAAATGACCT
LH08G:01247:00947	73	100	NZ_AJTT01000001	351245 to 351317	CTTGCCACCTGAATATCGTTGCAACTATAAGTAAGTTAATTATCGAACACATTAATTGCAGTTGATCAAAACT
LH08G:01297:00604	245	99.592	NZ_AJTT01000001	366937 to 367180	AATGTTTTTTAAGTTTTTTCTGAATACACTATAATTACATCATTTGTAACATTAAGCGAATTCTTTAATACCATTTCAAGCATTGGAACTCCACCGACTTTATGCATTACTTTCGGTAAATCGGACTCCATTCTAGTACCTTTACCGGCTGCTAAAATAATTATTTGATAATTTGCATCGTTATAAGTCATGGTTCTCCTTGTATTATTTCGTAAACATCATCAGATAACTCATAATTCCCAAAA

Read name	Sequence length	% ID	GenBank accession no.	nt coordinates	Sequence
LH08G:01318:02288	287	99.306	NZ_AJTT01000001	7990 to 8276	CTCTATTCATAATATCTGAAAAAGATCGTACAAATTCTGTTGCTCTAATAATTTGT GCCATATTTATCCTCAAAAATAATTACCATATTTATACCTTTGATACTTCAAGATT TGGTACGTCAAATTTCGGGATTCGCCTGTGCTCACTATTGTACGCTGTGTAGGC GCACTGCTTATTTTGACTTCCAACTTTGAAGTATCTACGGTATACATATTATATG ATTATGTGTAGATTATGTAAATATGTTTATGTAATATAAGCTAAAAATTCTCTGAT TAAGATTTGA
LH08G:01333:00336	374	97.861	NZ_AJTT01000001	258664 to 259030	TTATCTAAAGCACTCTTATCTTGAAATTCTCTAAAGCCCGCATTTGCTAAGATTT GACCTATTTTAGGCAAATCATCCGAATGTTTTTTTTAAAAAATTTCCCATATTGGA GATTTTTTTAAGAAGTGATTTTTGAAATTCCGGATCAAGATATATCTTAGAAGAG TTAGCAAGCTTAACAAGATTATAAGTATTTACAGGATTAGTTAAAAATAAGTCTT GCTCCGAGTAATATCGTTCTTAAGCTTATACTCTTTGAAGATGATAAGTCCTGAA TTGCACTCTTTAGTACTTTATTAGCTTTAGAAGAAGATAAAAATTTTTCTACAACT TTTTGATAGTGTTTAATTATTTATAACAACCATAAAATTCAC
LH08G:01354:00365	286	99.65	NZ_AJTT01000001	262020 to 262305	GAGGTTTTTGTAATATATCATCAAAAGCTTTTATATAGTCGCTTGGAAGTATTTCA GTTATGTAATTCGCAATATCGTTACCTTTTTCATTAAAAAATTTTCAGCAATTTTT ATCCGTGGATAACATTCCTAATGTTTTAGAAGTTACTTTATTATAGTCCGGTACT TCTTGCTTTATCTCAAGTGCAATTTTTTGAAGGCTTTTAAGTACAGCATCATTTTT AAAACATTCTTTTAGTATTTCGAGTCCCTTTTCATCTAAAGCACTCTTATCTTGAA ATTCTCT
LH08G:01360:02559	261	100	NZ_AJTT01000001	366624 to 366884	TCAATTATTTATTCAACCATTGCGGAGTTATTCAGTATCCAATAAATATAGCATCT AATGAAGATGTTTTAGAGGCAGTCATAGAAGCCGGGGGGCATGATATTATATCC GATGATACCACCCATACAATCTATACGGATATTGAAAATTTCTCTAAAGTTCTAG AATTTTTAACTGGCAAATACGGCATACCTGAAGATTCGTATATAGGCTGGATCC CATTAAATACAATAATTATAGATGATAAGGAAAAAGCTGAAA
LH08G:01418:00298	332	100	NZ_AJTT01000001	246549 to 246880	GATAGTTATATTATCGCCTATTTCTACCGGTCCGTTAAACCTTGAAGCATGAATT ACACTACCGTCTTGTATGTTAGTATTATTTCCTATTTTTATCGATTCAACGTCGCC TCTAAGAACCGTATTAAACCAAATGCTTGAATTACTACCTATTTCAACATCTCCT ATTAAAGAGCTGCTTTCGGCAATATATGCACTTTTATCGATTCTTGGCGTAACTC CTTTGTAAGGGATAATGAGCATTAATTATAATCTACCCAAGAAATATTACCGTCT TTACGATAATAAACTATATTGATACGATCATTATTTATATTTTTAAATACTACTGC
LH08G:01483:00337	349	99.143	NZ_AJTT01000001	117122 to 117469	TAGAAAAAATAATCATTGAACGTGTCAGATCAGTATCGATACCTTGTGAATAATA TTTAAATTTTCATTGACTTAGCATTACCAAAGTATTAATTTTTGTTAAGGTTTAGT AAATTCTGAACCGTCAACAATTTATTAAATATATTGGAGGAAGGTTATGAGTAAA AGTAAGGCTATTGAAAATAACGGTATTAGTAATACCAATAGTCCAATGGTAAATA TATGGCCCCAAGACCGGAAGGAGTAAAACCTACCTGTGTAGTAATTACTTATTC TGTAAGCAAGGATATTAAGGCTGTTCGTGAAGTATTGGATGAGCGAGGTGCAA GTGTTCATTACATAATTGATA
LH08G:01503:00952	368	98.113	NZ_AJTT01000001	22507 to 22873	ATAATAAGTTTATATGCTTCTGGATAAAATGACGTAATGGCATGATGTAACCCTA ACAAATTATAGCTGTTCTTAGATGCTAAAATTTGATTTTATTTCGTCATATTTTTTA GTTTTTATAAAATTTTTTTATTAGCTTATTCAAAGCTGTTGTGGGTCATTGTGAAT AATATTCAGATGGTAAAGACATGTTTTATATCCTATCAAGTTGCTATTATTTCATG GTAATATGTTTTATAATAAATAGTATATCTTTCTATTTGTAAAGAAATAATTTTAAT TACAAACGAACATTAACATCTTTATTAAGCTCTCTTATCTACCTCCCAAATCATTA TTAATCTGAAAACATAAATTTATTGGAAAAA
LH08G:01617:00382	252	100	NZ_AJTT01000001	183939 to 184190	ATATATGCTTCAGGAAGATATGCCTTAATATATTCCATATCAGATGCGTTTTTTAA TAAATAGGGTGATACAAATTCTAGTATGTCCTTACCGCTTCTTTTATAATTAGCA AATGAGTAAGCAACACAAATACCGTAGTCATTTAATAAATGCTTGTGTCTTGTCT

Read name	Sequence length	% ID	GenBank accession no.	nt coordinates	Sequence
					GTTGAATGTTGTTTTGCAGATTCCACAGCTCTTAAAGCATATAATATCTGTTCTC<br>TTGTTGCTTGAATAGAAACATCATCTGAAGC
LH08G:01673:02121	312	99.359	NZ_AJTT01000001	247493 to 247802	ATTAATAAATCTATCTTCTACTATTCATACTAATGATTTAATATCAAAAATCAATGA<br>GTTAAACCTTGATAACGAGATTTCAGGAATTATAGTTCAGCTTCCTTTGCCAAGC<br>TCCATAGATAAAAATAAAATTTTATCGGCAGTATCACCTTCTAAAGATATCGATG<br>GTTTCCACCCTTTAAATGTCGGTTACTTACATAGCGGAATTAGTCAAGGCTTTAT<br>TCCCTGCACTGCTCTTGGTTGTCTTGCAGCTATAAAAAAAATACGAACATAATTT<br>AACCGGTAAAAAACGTTGTAATTATTGGACGCTCA
LH08G:01752:01279	215	99.074	NZ_AJTT01000001	366307 to 366521	CACTAAATTAATTCGTGAAATAGTTACTGCAGCTAAAACCGGTTCTTCTAATAAC<br>CCTGAAAATAATCCACGTCTTAGAAATGCTTTAACCGCTGCACGTAGTCAAAAT<br>CTCCCTAAAGAAAGAATAGATAAAGCTATTAATAGTGCGAATGATTCTTCTAATA<br>ACGAAAATTATACAAGAAATTAGATATGAAGGTTATGCACCGAATGTATTG
LH08G:01910:02516	322	97.059	NZ_AJTT01000001	138157 to 138477	TCTATATTATTTTTCTCTGCCCAAATATTAGCAAGATAAAGAAGTGCTAACGAAT<br>CGCTCCCACCAGATACTGCAATCGATATTTTAGATAAGCCAAAATTACCTATTAG<br>ATTATTGATATTATACTCAAATTTTTCATATAGCATGGTTGAAAAATGTTAGTATT<br>GTAAAGTGTTTGGATATTGTGGTCAAACTTAGTAGTATGTCATTTCCGCGTAGG<br>CGGGACGTTGTTGCATGGCTCTTATGTCATTCCTGCGAAAGCAGGAATCCAGA<br>AAAAAAAGTATAAATACAGCAAATTCTTGAAATTAAAAGCTCGATTTAT
LH08G:02102:01594	285	99.298	NZ_AJTT01000001	359185 to 359467	GAAAGCTTTACTTAATTCTTCCGAAAAATATGAAGCAGCTCTAAAACATTTTGAG<br>AATATAGGTATTACTGCGGATGTAAAACTAGACTTACAAAAAAATGCTAGCTAAT<br>AAAGATAAAGTAGTTCTTGATCTCACAAAAGGTATAGAAAGTCTCTTTGCCAAAA<br>ACAAAGTTACTAGGATAAAAGGTGAAGCTAAAATTAGCTCTAGTAATAATCGTTG<br>AAGTAAATAAAGAGCAAATTAAAGCCAAAAATATCCTAATTACTACAGGTTCCAG<br>CGTCATAGAA
NB501781:2:HGMVWAFXX:3:21506:4952:12115_1:N:0:TAGGCATG+ATAGAGAG	149	99.32	NZ_AJTT01000001	252942 to 253088	GTATTAAACGGTTCTATTTGTGACGAGAATGAGGAGAATAATAAATGCTGTCATT<br>TTAGCGTACCGGTTAATAGAGATTTCAATGACCCTGTAATTGATTTAGGAGAAG<br>CAATATGCAAATGAAGACTTTATTGTTACTCCTGTGTGTA
NB501781:2:HGMVWAFXX:4:21504:24946:16382_1:N:0:TAGGCATG+ATAGAGAG	151	100	NZ_AJTT01000001	198785 to 198935	GCCAAAGAACGGTGGGAAGAAATAATTTTTGACTAATAGCTAAAGCTTCTCTATT<br>ATTTCCCTTAAGCACACTAATTAAATATTGGTTATGAAGAAAATTATCGGATTATT<br>TTTTGTAATTATACTTAGTGCAATAAGTACTAGTATCTTA
700666F:214:CA2UUANXX:8:1207:11637:88986_1:N:0:TAGGCATG+CTCTCTAT_(reversed)	125	100	NZ_AJTT01000002	94923 to 95047	GAATTATGAAATATCATTTTTTCAAAAGTAGGGCATTAAGCAACTCGCTTAGTTG<br>CTCTAAATGTCTTTAAGTACATGCTTTAAGTATGTGTTTTCACATTACTAGAAAAA<br>CAGAGGGCTTAAGT
LH08G:00117:01579	305	99.016	NZ_AJTT01000002	112641 to 112942	ATATGGTGCTAATCGGATATACTCGATCCTGACGGTCAAATGGTCCATGACAAG<br>GAAGCAATTTATGAACCTCTGAAAGCTCTTTTTAACGCTTGAATCCACATTGCGT<br>AAGAATGATTACAAGAAAATTAAGGCTATTGACAAGAAAGATTTAGCTGCATTTG<br>TTCAATGGAAAGCAGCAATATTACCGGTTGTAAAAGATGTTTCTAAGGATGAGT<br>ATATAAAAAAGCCTATTAGAACTATATAATATTTTCAAATCAAATGAGTTTGGATT<br>CGGATGATTCTAATAAAACAACCATTAATAG
LH08G:00213:00620	202	97.03	NZ_AJTT01000002	189029 to 189225	TCCTAACTCTAATAAAAAAGTTTTTAATATTATCTATTAATTTATTTTCTAAATCTC<br>GTTCTATTATTTTACCTTGAATATCTAAAAATTCTAAGTTATATGGTTCTTTTTATA<br>ATCGACCTGGCTAAATCGGACTGTAGCTCAGGTAAAGTATTGGAAAAGTTATTT<br>TATACCTTTCGCTGAGCTTTTCATGTTAAATTAC
LH08G:00276:02094	303	99.67	NZ_AJTT01000002	114637 to 114938	TGGGTATAATTATAAAGTTCATTTAGTATTTTCCCTTTTATGACGGGCATAGTCTT<br>GAGATACAAATAATCATATGAACGATATGCTATATTAATACTCTCCCACCAAAGA<br>GCATAAAATACTGCCCAGTAAATCATGGAAGATAAAAGATTATTAGCGTTCTTAT<br>CGCTGAAAGATTCTATTAAATCAATGATTTTCTGAAGTAATATACTATCAATCGC

Read name	Sequence length	% ID	GenBank accession no.	nt coordinates	Sequence
					CGGAATCATACCGAGTAAAATACAAATTACAGTCAATTAAAGTCATTTTAACTTT<br>ATCGAATTTTAAAAAATAAATACTTAA
LH08G:00319:00921	308	99.676	NZ_AJTT01000002	145726 to 146034	AAAATATATTTTATATGGATTGGTTAGAGAGCTAAAATTTGCGTTTGAAGATATAA<br>AAATTCATGATTTTCAATGCCGGAAACTCAAATTGATACGGTTAATTGGGAAACC<br>GAAACAACAACAAATTACTGTCTAAGTAATTTCATTATAAACTTATTTCATATAAG<br>AGAAGTAAAAGATTTTATTGCTAATATAATCTAGCAATGCATTTACTGCACATATA<br>ATCGGTGATAATAATTATAACCTAATAAAGAATGATAGTACGTATTATATGATTGA<br>ATCTAGTTGCGATAAAGATTAGTTTATTG
LH08G:00347:00812	376	98.143	NZ_AJTT01000002	168719 to 169090	GAAATGTCCTTCTAATACCGTCGAAGATACGGAAAAGAATGCAGAGTCTTGATT<br>TGAGGGCAATATGTCATTCCTGCGAAAGCAGGAATCCAGTATCCTTAATGTCAC<br>CCCCGTGGGCTTGACCACGGGGGGCAAAATAATAAAAAAATACTAATAATATTA<br>GTATTTTTTAGCTGGATCCCCGTGATCAAGTCGCGGGATGATAAATGATAAACC<br>TATCCACGCCACAAGGCCTACGCGGGGATGACATAGCAATGCTGTAATGACGC<br>GATAAAAAACATAAACTTAAAATAAAAGTGCAGAAAAGTTTAATTACAAAATGGT<br>TGTGTATAAGTTGTATAATGGTCATAGCGACGATTGTTATAGGGGGGATAAC
LH08G:00422:02472	293	99.317	NZ_AJTT01000002	197112 to 197402	TAAATAGTTGCCCTCTTAGTTCTGTGTTAATTTCTGCTGGATAAATAGAAATAGT<br>GGCTATTTTCTTTAAATTATCCTCACCAAATTTATATATTAGATGATCACTGAAAA<br>TACTTCTTTTTTCTAACAACATTAAGAACTTATCTTTATTATCAGCTGCATGAGAT<br>AAAAGATATTTTTCAGTTGTAATACTACTTACGTAATTAATATCACTTGCTGCTAT<br>TTTAAGAGCTAATCCTTTGTTTAGGAATAGAGGTTTATTTTTATATTGTTCTGCTT<br>AACGGATCGTCTGT
LH08G:00512:02380	367	99.728	NZ_AJTT01000002	42143 to 42508	GTTTGAATAGTAAACTCGATAGGGCTACTTGCATTCCTGCTAACCATTGAACGT<br>GGATCCATAGCGTAAATCGACATGCCCGGTATTTCAGAAAACTGCTTATTTAAC<br>ATATTCTTAATAGTTTCTTGGGAACAAGAACGCTCACCCCAATTTTTTAGAGGAA<br>TGAATCCAAAAACGTTATCGCTACCGCCTGCACCTATTACCATCAGATAACCTA<br>ATATATCTTTATAATTAGCAAGGATTTTTTCGGCTTCTTTCACTACTTTAGTAGAA<br>GATTTCTAAATTAGAACCTTCAGGACCTTTAAGAGAAACTTGTAAGCATCCATCA<br>TCTTCTTGGGGTACGAAAATGTTTTGAGTAAATTTAAAA
LH08G:00520:01570	270	99.248	NZ_AJTT01000002	90561 to 90825	AGCTGATTTTAAGCAAGAACGGCAAATAGCAGTCGGTATCGATTTTGGCACTAC<br>TAACTCATTAATAGCTATTGCAGCTAATAGACAAGTTACAGTGATTAAATCTATA<br>GATGATAAAGAATTAATCCCAACTACTATAGATTTTACAAGTAACAATTTTACTAT<br>AGGTAATAATAAAGGACTGCGTTCTATTAAAAGACTATTCGGTAAAACATTAAAA<br>GAAATTCTAAATACTCCGGCATCTTTTTTCGTTAGTTAAAGTTATATCAT
LH08G:00565:02879	201	100	NZ_AJTT01000002	101209 to 101409	GACATTGCACTAAATCCACTTATAGACACTGGCATCATATTACTAAGGCTAACTA<br>TGCATTCCTTAACTCGACAGTTACTTAAAATAAAATAAGCAAGAAAGATATAACC<br>AAACTGAGCAAATACGATCGTCACAAAAATGAACATATAATCTTTAATAATTATAT<br>CTAATACTTCATCAAACTGTAGTTTTACGATAAAG
LH08G:00664:02910	259	100	NZ_AJTT01000002	135550 to 135808	TAATTTTTGAGTGGCATTTTTACGGGCAATATTTTTTTCGTTTTTCTTACTGATTC<br>TAAACTCAGCAATGCTAATTACATGCTCAAGTAAATTAGTAGCTAAAGCTTTATC<br>GCTACTAAGAAAATGGTCAAAATGATCTTTTATTATATTCTCGACCGGTTTACTT<br>ACACCGTTTGATACTAATTTTTCCTTAGTTTGTCCTTGAAAAGACGGCTCAGCTA<br>TAAAAATAGAAAGTACGACACTTGCGGTTTCTAAAATA
LH08G:00685:00592	312	98.718	NZ_AJTT01000002	80051 to 80358	CGAGTGGCTCACGATTCACCCACAAGATATTATTTATAATAAGTTAATTAGTTTG<br>CCGGTTAAATTTAATCAAAATAAAAAAGCTTCCTTTTTATTGCACCGCATTTTTAC<br>TTTTAGACATATTAGCAAATAATGATAGCCCTATTATACGCACTACTATAGATAAA<br>AATCTGCAAGCTGCTATAGAACAACAAGTTCGGCTATATATTAATGATCGGACA<br>AAATATGGCATTAATAATGCCTCAGTAATTCTTATTGATTTTACTACTATGGAAGT<br>GCTTGCAAGTATTGGCTCCGGAGAGTTTTTTAATA

Read name	Sequence length	% ID	GenBank accession no.	nt coordinates	Sequence
LH08G:00800:02828	356	98.603	NZ_AJTT01000002	76876 to 77230	TGTTTGTTGAATTGCGTTATTATTTGGGTTTCCTAAGAATATTGTATGCCATAAG CATTGCATCTCGTTGTTTTTTGAAAGCAAAGTTCGTTTCCTTGGGCCCATATATA AATCATATTTTATAGGCTTTTCAGCCTTATTTATAAGACTGTCATAACATTTTTAA TATAGAGTCTAATTTTATTATTCTTTCTATAAAATATGATGCTGGGTCTTCACGTA CCTAGTTAAATAGCGTATCGTCGATTTTATTGTACATTTCTTTAAGTTCTTCATTA CATGTTAAGCTGCATTATAATTAACTAAAGCTTCATTATAATCTTCTGCTTCGCTC AATATATCGCCTATTTTAGAGT
LH08G:00811:02060	260	99.615	NZ_AJTT01000002	173770 to 174028	GCTTTATGGGGTTTGCCGGTAGTTTATATTATTGAAAATAACGAATATTCGATGG GGACGTCTGTAGCACGTTCTACCTTTATGCGTGATTTATATAAGAAAGGGGCAT CGTTTGGGATTAAAGGATTTCAGTTAGACGGTATGGATTTTGAAGAAATGTATG ACGGCTCTAAGCAAGCAGCCGAGTATGTTAGAGAAAATAGCTTTCCTCTGATAT TAGAGGTAAAAACTTATCGTTATCGTGGGCATTCGACTGTCCG
LH08G:00904:02287	276	98.551	NZ_AJTT01000002	170805 to 171077	AAATATGGTAATAAAGAAATAATGCCGTATAGTAAATATATGTTCTTGCATGCTA ATAATATATGTTTATCTGAAAAAAATTTTTTGGTCAAGAGATTAAGTTAGAAGCAA AATTACCGTTTTATTTTAATAGACGTCTTACATAACTTGCTTCTAAAGGTAATTTG TACGTCGATCCGGTACTCGAGTCCTCATGTTAAGTGTACGCTGTGGTTCTGCGT TCCGTGTCTCCTTCAAATTTCTTTTTTATTAGCTGCGTTATGTAAGACGTCTATT
LH08G:00929:00391	262	100	NZ_AJTT01000002	196215 to 196476	TAACATTAGCAATATTTAGTATATTGAAAGAAGTATTAAACGATTCCGGCAACGA AGTCAAAATTGTAGTTATTTGGAGCTTAACTGAAACAGTACGTATAAATCCTAGT TTAGCCCAAGAGACATTAAAAATATTGAATACACTACTTAATAACCCTAGTAATT ATATTGAATTCACAATCGCCAAAATCCTAGGTTGGATAATACAAATAAACCCCAA TATATCACATGACGCATCTAAAATACTGAAAAATTTATTTAG
LH08G:01004:01941	335	99.403	NZ_AJTT01000002	82901 to 83234	CTAAAAATAATGGAAATCCCGGAAAGCTAGAAGTAAGAACATTAGATTAGGTGC AGAAACAGATTTAGGGATTACTATCAAAACACTTGTAACTTTAGAAGAAAGGAAA AGTTCACTTGAGTCTTTAGCATTATTTTCAGAAGCAAAGCATGATAAAGCACAAT TAATGATAAAAGCTGAGAATATTTGGAATAGACTAAAAGATGATGATGTAATAAA GCAAATTTCAAGGCAAGCTTTAACTTTAAATAATAAAATAGCCAACAAGGAATTT TTTTAAAGAGCTATTTAATGAAGAAGAACATAATGCAATAGACCGTTAAACTATA CTTTTG
LH08G:01195:02415	201	100	NZ_AJTT01000002	127231 to 127431	TAAAGCCGGTGAGAAAGTGGGTATTGTCGGGAATTCATTAGGTAGTGGTAAATC CACTTTAATTGCATTATTATTGAAAAATTTTAAGCCGGAATTTGCGATATTATAAT CGATAACCAAAGCCTTTACAATACTTCCTTTGACAGCCTGTGGGAGCAGATATC ATTAATTCCTCAAGATATTATGCTTTTTCATCGCTCA
LH08G:01210:01821	157	97.452	NZ_AJTT01000002	114802 to 114954	TAAATCAACATTTAGCTTAAGTATTTATTTTTTAAAATTCGATAAAGTTAAAATGA CTTTATTTGACTGTAATTTGTATTTTATCTCGGTATGATTCCGGCGATTGATAGTA TATTACTTCAAGAAAATCATTGATTTTAATAGAATCTTTCAGCGA
LH08G:01267:00554	256	99.609	NZ_AJTT01000002	84100 to 84354	ATTCAGTTGACACGGATTTCAAAAGAGAGTTAGCAATAAAGACTTAAGGAAAGG AAAAAACTAAAAGCATTAATTGTAGAAGAAGTTTTTGAGAAATATACAACTAAAA ATGCAGATGCACTTGATGGTAATATAAATCAATTAGCTCAAGCTATAAAAGAAGG CACCAATATTGAGTTCATAGGTCCTAAAAGAGAAGTAGCAAGTGGTATAGATCA GGAAACAAGAAATCTATGTGTTAGAATGAATCAAGATG
LH08G:01288:02068	234	99.145	NZ_AJTT01000002	65637 to 65868	TTTCTTCAGCAGGTTGGCTCTCGCTTTGTGCTTTATACATCGCTTCACCAATTTT CATGCTAGCTGCAGTTAAACTTTCGGTCTTTTTCTTTAATCAAAGCAGTATCTTC CGACTCAAGCACGGCTTTTAAAGCGGCTAGTGCTTCTTCAACAGCTCCTTTATC GTCTGATGATAATTTATCGCCATATTCTGTAAGAGTTTTTTTCAGTAGAATAAACT AAACTATCGGCAGC
LH08G:01422:02299	310	100	NZ_AJTT01000002	157879 to 158188	CCGATTCTCTTAATCTCCCACTCTAACTTAACCCCGCTATCTTCATATACTTTCT GCCGGACGAAATCACCTAAATCTTCTAAATCCTTAGCGGTAGCATCACCATTAT

Read name	Sequence length	% ID	GenBank accession no.	nt coordinates	Sequence
					TTATCATAAAATTACAATGAAGTTCTGACATCGAAGCACCGCCTATTCTATAGCCTCTAAGCCCCGCTTTGTCAATAAGTTCCCAAGATTTACGCCCTTCAGGATTTGCAAAAGTACTACCGCCCGTACGCTCTTTAATAGGCTGTGTAGATGATCTTGCGTTATTTATTTCATTCATCCGTAATAATATATTTTCACTAT
LH08G:01567:00542	283	98.233	NZ_AJTT01000002	25976 to 26253	TCAGTGAATTTACTTGATAGCGTAACCCGTGGAAGCTTACGGGAGTAAAATACCGCTTTCACAAGTTGCTTCTCTATCCACACCTGATGCACGAACAATTAACGTGCAGGTTTGGGATAAATCTATGGTATCATCGGTAGAGAAAGGAATTACGATAGCAAATCTTGGCTTAACTCCTGCAACGGACGGTCAATTAATCAGACTGCCGATACCGGTTTTAAACTGAAGAAAGACGTAAAGAACTGGTAAAACTTGCTCAATAAATACGGTGAAAGTATACTAA
LH08G:01579:01219	245	100	NZ_AJTT01000002	98497 to 98741	GTAATTGCAGCTTTCGTTAGTTTTAGGGAAGTAGTAATTACTAACCGTACTATATATTTACTCAATAAAGCAAGAGATAGAGCTCATATTTTACTAGGACTAACTATTGCCGTTAGTAATATCGATGAAATAATACGTATTATTAAAACTTCAAATGACCCGAATGCAGCTAAACAAGAATTAATGGCTCGTCAGTGGGAAGCATTAAATATCCTACCGCTTGTAAAGTTGGTTGATGATAAAGC
LH08G:01648:02712	190	98.936	NZ_AJTT01000002	188231 to 188416	TCCCTGCTAATGAAAAAAATCTGCAAGACATTCTTATATCAAAATACCTATCAATTCCGATACTCAAAATATAGAGGCTGCAGATAAAGAGGACGGATATAATAGGATGAGGCTTGGCATAGATAGAGATTGCAAGAGCAGTAGCAACTAAAAAAACTTGATAAGCAAATATCTAGCCTAACATATGAAG
LH08G:01694:00924	330	99.698	NZ_AJTT01000002	125784 to 126114	TTTCATATCATACCTGCGTGCCCTTGTTGCATGACTCTTATGTCTTCCCGCAAAAGCGGGAATCCAGTACCTTAAAACTTTATTTGAAAAGTAAAATTTATAAAAAAAATCTTGGTCTAAAAGATTTTTTTGGAAAGCTCGATTTATCTCGCTTTACCCTGGATTCCCACCTTCGCGAGAATGACATCGATCCTTAAGTTGTCACCCATATGCGATCAAGGGAATGACATTATTGTAGATAATCTATTAATATATATTAGTATCAAAGGTAGCCGTTCCACTCATTACATAATTTAGCCTGATATTCTACACTATCTAGATTTTTATCTTTC
LH08G:01766:00833	390	99.235	NZ_AJTT01000002	55480 to 55870	AATAACTTTTCAGGATTCTCAATAAGCTGCTTAATTTTTACCAAGAACGATACTCCTTCTTTTCCGTCAATTATACGATGATCGTAAGATAAAGCTATATACATCATCGGACGTATTTCAATTTTACCGTCTATAACTACAGCTCTTTCCTCGGTTTTATGTAAGCCTAGAATACCTGATTGAGGAGGATTAATATCGGTGTAGACAATAATGAGCCATATACGCCTCCGTTAGAAATCGAGAATGTCCCACCTGACAAATCAGCCATAGAAAGCTTAACCCTCACGAGCTTGTTTAGCCAAAATTCCTATAGCTTTCTCCACTTCGGCAAATCCCATTTTATCGGCATCCCTAACAACCGGTACAACAGCCCTTGCGCTGTTCCGACAG
LH08G:01773:01731	203	100	NZ_AJTT01000002	116592 to 116794	ATAGGTATTGTGGGGTTATATCTTTACTTGAAGGAAAGATTATATACTACGCATTGGTTTCAATATTGGTATATATTAATGTCACCTTCAGGTTTTATTGCAGTACTTGCCGGTTGGTTCGTAACCGAGGTAGGTAGACAACCTTATATAGTATATAATATTTTAAAAACCGTGGATACGGTATCACCGGTACTCGGCAAATA
LH08G:01821:03034	311	99.045	NZ_AJTT01000002	154963 to 155276	TTTTATAATTGATCAAATTGTTTTAGAAATGACTGTTAATGAACTTGTCCGCAACCCAAACTTAAAGTTAGCCGTTAATATTTCAAATATAGGAACAGGCGTGAAGCTTTATGGGAAATAGCAGAAAACTTATTAAAAGCTCATAATGTTCGGGATCGTTTAATTATTGAAATTACGGAAACTTCTTTCAATCAGCATTACGATAAAATAACTTTATTTATTAATAAACTACGTAAGTACGGATGTAAATTGCATTAGACGATTTTGGTGCGGGTTTTACTTCGTTTAAACAACTTCAAAGCTTACCATTG
LH08G:01889:02706	393	98.728	NZ_AJTT01000002	109861 to 110249	GAAGAATTAACCACACCTAAAATAATAATACATTATCAGAACTTTATGCAGGAAATTCATAAATCTATGCATAAATTACAAACAGCAAATCAGAAAGATATCATCCCTAAAATTGCTAAAAATTATAACTAAACAAACTAAAGTGTTATGTATAGATGAGTTTGAAATTAAAGATATTACTGATGCAATGATAATCGGTAGATTATTTAATGAATTAATAAAGCAAAATATTTTTATTTTTATAACTTCTAACACGAGTCCCAATAATCTGTACAAAG

Read name	Sequence length	% ID	GenBank accession no.	nt coordinates	Sequence
					ACGGATTACAACGAGAATCTTTCTTACCTTTTATAAAGATAATCAATAATACATTTTATGTTAAATATCTAGACTAATTCAACATGAATTATCGATTTGATAAAGCGTTAATAGT
LH08G:01959:01839	372	99.194	NZ_AJTT01000002	127037 to 127405	CTATGGAACTTAAAGACTTTTTAGAAGATATAGCAGCGTTTCGCTCCACCTTTACAATCATGCAAATCCCACAAGATACTGTAGATAAAAAATGCTGCTGAATTAAAAATCTTCAAAGGGGAGATTATATTTAAGGATATATCGTTTGCTTATAAAGAGGGGAGTAGTGTATTCCAGTCTCTTAATTTGTATATTAAAGCCGGTGAGAAAGTGGGTATTGTCGGGAATTCATTAGGTAGTGGTAAATCCACTTTAATTGCATTATTATTGAAAAATTTTTAAGCCGGAATTTGCGATATTATAATCGATAACCAAAGCCTTTACAATACTTTCCTTTGACAGCCTGTGGGAGCAGATATCATTTAATTCCTC
LH08G:01985:01148	269	98.507	NZ_AJTT01000002	13347 to 13610	TTTTTTATATTGATCAACAAGCTTATTAACTTTTTCTTGTTCTTCCGGTAGTTTTGAAATATTTTCAGCAATATTTGGCATTTGTTTCTCTTCTCTTATAAAGTTAAAACATATTAATTTACATTATATATATATATTTTTTATATATTTTTTTTATACAAGAAAAAACTTTAATAGAAATTAACCTTTGAACGCAGCCTTAATACCTAAGAAAGTCTAAAAAATTTCGTAATTCTTGACGTGCAAGCACGTGTGAGCTATTAAATGCTCT
LH08G:02060:02022	249	100	NZ_AJTT01000002	38275 to 38523	TAGCCACCATTATGTTTGTAACCAACGCCATCTCCCGTTAAACTTAAGGTTGGTACGCGAGTTATTGCATTAGATATTATCTTAGCGTTTGTTCCTTCAATATATTTACTGGTGAATTTATCTATACCATTTTTATCTATAGCAGTACCGTATACAAAACTTCCTAACCCCGAAGTAGCCAAATACTCTTCAACTTTAGTTGCTTCATGAGCTTGAGTAAAAAGCTTTATTTGTTCATTACTCTCCATA
700666F:214:CA2UUANXX:8:1212:1167:48524_1:N:0:TAGGCATG+CTCTCTAT	125	100	NZ_AJTT01000003	10090 to 10214	AGGTAATTTATGTGCGTGGATCGCAGGAATCCAAAAACATTTAATGCAACCCCGTGGCTTGACCACGGGGTCTAGAAAAACAAATTATTATATTAATAATTTTAGTATTTTTAACTAGATTCCGC
LH08G:00104:01732	234	98.298	NZ_AJTT01000003	94226 to 94457	ACATATAAAGACCTGCATACAAAGGACTTAACCCTTTGATAAACTGAAGGACATAAGATACATTATGGATCCTAAGACACACGTTAATCAGCAAATAAACTATTACTCCTATACTAAAATTAGCTAATTAAGAATAAACGCATATCCACTAACGGATCATCAATTTAAGTTGGCGTAACGTAAGGTAACTATTATTGAGTAACACAATGCCAATTAATAGCATTCCGCCGGATG
LH08G:00249:01567	320	98.438	NZ_AJTT01000003	60594 to 60908	CATATCATTATTGAAGTTTCATATAATAAAAGTAGCGGTATTGCAAGAGCAAACTGGCTTAAAATGTCAGGAGGCGTTAATATTCCTGCAATAATAAAGTTAATTACCACGGCAATGCATCTTTTTTTGCTTTAAGTGTTTGTACCTTAACTATTTTTAGTATATTTAATATCATTATCACAACTGGCAGCTGAAAGGCGACTCCAAAAGCAATAATTAAATGAATAACTAAATTTAGATACTCGCTAATTCTTGCTTCCAAAACTATCGGTACGATCATATCACTGTTTTTACAAAACATAAAGAAAAAAATTCCAAGCT
LH08G:00430:02505	386	98.187	NZ_AJTT01000003	67256 to 67636	AGTAAGACAATAAACTTTAACATTAATTTAAAATGCGACCTGAATTTATTCATTTAAGAACTCAAAGCTCTTATTCTTTTTCTAGAAAGTGCATTAACGATTGAGAAGGTAGTAGAACTTGCTTCATCGAATAAAATGCCTGCTATTTGTTTGGCAGATAAGGGAAATTTATTCGGCTCATTAGAGTTTGCATTATGTGCAGTAAAAAAAGGACTGCAGCCGATACACGGAGTTATTCTAAATATAAAATACGATATAGATATTTTTGCTACAATTTTTGTCTTATCGCTAAAGATGAAACTGGTTATAAAAATTTACTCAAATTATCAAGTCTTACTTTTTACTAAAAAATGATCGTAAGATATGCGATCACATTGCTTTTGAAG
LH08G:00524:01912	296	96.939	NZ_AJTT01000003	123597 to 123881	TGAAGAAATTGTTACAAAAACAAAAAATGCTTGAATTTTTAAATGAATATGAAAACCCTTTTAGCTGAGATTTACAAATAGGACATTTTCACTGCTTCGGCTTTTTTTACTTAATAATGATAAAACTAAATTTTTATTGATGCAACCATAAAAAGCTTGATAAGTGGCTTCAACCAGGTGGTCATTGTGATGGAGATAGTAATATATTAAATGTAGCGGTTAAAGAAGCTATAGAAGAATCCGGAATTAATGAAATCAAAACGATAAAATTAAGGAGAATTTTTTTGAATATAGAAC

Read name	Sequence length	% ID	GenBank accession no.	nt coordinates	Sequence
LH08G:00803:02840	351	97.721	NZ_AJTT01000003	95748 to 96090	GATAGTCAATATCTGTGTAACAAGGATTAAATATCTCAAATATTTCTATAATGCTTTCTTTATATTCTTCAGACTTGGGATTTGCTGTTCGTATAATTGTTATTAAGCGTGCAAAGTTGCTGATAGTCTGAAAACTATCATCCACCGCCAAGACTCTTACATATTTTCATGCAATATTCCGCTTGAAGAATCTAAGTTTTATTGCCTATAGCGGATGGGGAATTTAATTTTACAATTTCATTATTTAAATTTAGTTAGTGGTAGTATTGAGAATTCATATTCCTTAATTAGATTTTGCTAGTTTTATAATATCATGTAAATTATTTTATTTTTTATAACTTCATCGAATAT
LH08G:00830:01910	224	98.661	NZ_AJTT01000003	133965 to 134185	GCTGTTGTTTTTCTTGTTCTTCCAATAGTTTTCTTTTTTCTTCCTCTACTTGTTGTGTTTCTAGTTCTTGCTTTTGCTGTTCTTCATATTTCTTTTCTGCTTTTTTTTGGTTATAGTTTTTATTTCTGTACAGATATTCATTAATTATTTTTAAATGTTGCCTCGCCTATGTTGTGCCAAGGAAGATAAAGCGACGTTAAAGATTGATTACCGCTTAAGCTTGT
LH08G:00869:03108	311	99.678	NZ_AJTT01000003	154896 to 155205	GGAGTATCAAACGTTGTTGTATCATTAGGACGTTTATCAAAATCAACTCCCATGATTTTAGGAATAACGTCACCAGCACGTTGCAGGAATACATAATCCTTAATCCGCAAATCTTTTCGTGCAATTTCTTGGAAATTATGTAATGTTGCTCTGCTAACAGTTACCCCCGCCTATTTCTATGGGTTCAAGTTCGGCTACCGGCGTTAACGTACCAGTTCTACCAATCTGTACAGTAATAGAAAGTAACTTTGTTTGTCCTATTATTGCAGGAAATTTATGAGCGGTAGCAAAGCGTGGACTTCGTGCAATAA
LH08G:00948:00904	306	99.673	NZ_AJTT01000003	84836 to 85140	TTTTAAAATGTTTTATTATTGATGAAAGGAATTGTTTTCTAAGCATTTAAATTAGTACCATGAAAATTAGAAACATAACGTCTAATTCTCAGTATAGAAAGAAAAAACGAACAAAGTCAAAACGCTTTGTCTCGTGCTGCAACTATAATCGCATGTATTTTTTTAAGCGTCCGGTCAAAATCATCGTTAGTAACTACATAATCATACTCATTAGAATGTGATATTTCGTTTTGTGCCGACTGCATACGTAATTTTATTGCTTCTTCATTATCGGTCGCTCTATTTCTTAAGCGTTGTTCCAATACT
LH08G:00952:02658	237	99.578	NZ_AJTT01000003	86156 to 86392	GAAGCTATGCAAGCAAGTAGTCAAATCCACCAATATAATCAAGATTTAGGTAATTGTGTGGATAATACTTCCAAAGATATCAAACTTACCGGTAAAGCTTCTAAAGGTGTATGTGAAATAATATAACATTCTAAAAGCTGTAACTATATAGTTGCAGCTTTTGTATCATTTTGAATTAAGCTCGAAAGACCTATTGCCTGTATTTTGTCTGACAATTTAAAGCTAATCTCACCTGGA
LH08G:01087:02301	176	100	NZ_AJTT01000003	47693 to 47868	TACTTGCGCTAATTTTTTGTCTATTACCCTTACGAATAACCCGTTTATTATGCTAGATGCAAGCGGTAACATGATGATCATTATGCAAATATTTTGATACATCATTATCCCCACATCTGATTAATGTCGGTAATTTTAATGGAGCCTTTATTACGGAAATATATTAGTAATATTGC
LH08G:01116:01184	207	99.01	NZ_AJTT01000003	29085 to 29284	TCTAGGGTTTTAAAAGGCTTTAAGCCTGACGGTAAACAAATTTGGACTTCTAAAGAAGCTATGATACCGCAGCATGTGCCGAAATCTATGATTATTGTCGGCTCAGGTGCAATCGGTATAGAATTCGCTTCGTTTTATAATAGTATTTGGTGTGGATGTTACCGTAATTGAGGCACATAATAGAATTATTGTCTGCTGAAGAATTAT
LH08G:01471:00458	132	99.213	NZ_AJTT01000003	45049 to 45174	TATATGCGGCTCTTAAATTCTTCTTATACACGTTCTTTGGTTCTGTATTTTTCTTACTTTCATTAATTTATATTTATAGCAAAATACATAGCTTTGGATTTAACCTATATCTTTCAGCTTACCGATATAGTT
LH08G:01892:02986	248	99.194	NZ_AJTT01000003	116847 to 117093	AAATAATTGGTAATTTATAAGATTTACTATAACTTTTATTTAAGGTTAAAATAATAATCGCAATACTCTTTAATAGGGCAGATGTCGCAATTGGGTTTTCTAGCTTTGCATATATATCTGCCGTGTAGAATTAGCCAGTGATGGGCATGCGTTAGCCATTTTTTATCGATAATCTGCAGTAGCTCTTTTTTCTACTATTTCAGGAGTACCGCCTTTAGCAAGCCCGATTCTGTTGGCTACTCTAAAGA
NB501781:2:HGMVWAFXX:2:11108:16748:12433_1:N:0:TAGGCATG+ATAGAGAG	151	100	NZ_AJTT01000003	42082 to 42232	TCATTGCGAGGAAATTACGAAGTAATTGACGAAGCAATCTCAGGATATTTGACGAGATTGCCACGCAGCCTACGGCTGCTCGCAATGACAACTCGGTATCCATGCGGGTGATGCCCCGCAGAAATACATCGATCTTTAATATATAATTTTT

Read name	Sequence length	% ID	GenBank accession no.	nt coordinates	Sequence
NB501781:2:HGMVWAFXX:2:21106:13745:11822_1:N:0:TAGGCATG+ATAGAGAG	151	100	NZ_AJTT01000003	1055 to 1205	TTCTTAGATTTTAATGCAAAAAACGGTACGGTAACATTAAATAACAATGTAAATGTTGCGGGAACAGTCCAAAATACCGGCGGTACTAATAACGGTACGTTAATAGTTTTAGGTGCAAGTAATCTTAATAGAGTAAACGGGATTGCTATGT
700666F:214:CA2UUANXX:8:1105:6238:13850_1:N:0:TAGGCATG+CTCTCTAT	125	100	NZ_AJTT01000004	123325 to 123449	CCTAATAGATTATCAAACGATACTCAAATAGTACCGGGACTACATGCTTCTGTAGAAAATAGCTTTAGCAATAAGCAACCTAAGGTGAAAGCTAGATTTATTTGGGCAGATAATTACTTTGAGAA
700666F:214:CA2UUANXX:8:2114:8166:42837_1:N:0:TAGGCATG+CTCTCTAT	125	100	NZ_AJTT01000004	70849 to 70973	CAATTAAATATTATAAATTACACTTTATAAATTATGTAACTTGTCTATAATAACTAATTAGTAATTAAGTTATTTAAAGTTCGATGAATAATAGGTCTAAGTTCATTTTTTTATCTGCTATTTCA
LH08G:00129:00502	269	100	NZ_AJTT01000004	64050 to 64318	GCAAAATCCGGTATAGTTATAAATCTTATCATATTAATTCAGATTTATATTTCAATATTAATGCATCTAAAATTGCTATGGTTTCAGCATCCAAATCGCCAGAAAAATTACTAGGACGAAAATGCATTTGAAACGAAATTATAATATCTTTCATTTTAGAGTCTAAAATTTTGGTAACTTCAAGTTTATAACCGTATGTAATAAATTTTTGCTGAATTGCTGCAATATCGGTTATGTCGACTTGCGGCAATAGATCGTTAAACACCTGT
LH08G:00174:00921	272	100	NZ_AJTT01000004	154704 to 154975	TGAACTAAATCAGGGTAAGGGTAAAGAACAAACGCAAATAATGCAAAATACCCTAAGAAAAATGAAGTAATAACATAAAACACGTTTTCTTGCTTTAAAATATCGCAGAGCTTAACATAAATTACCATAGCGATTATGGCAGAAGGTAGTACTATATATGTTTTTAAGAAACTTATTGCTTCTGCTCCTATATCTGTTACTACAAACCCGTCTTTAATTGAACGAAGCGTTGAGTAGTTTAATAAAATACAGAACATCATAAACGCCATAGG
LH08G:00219:01406	195	99.487	NZ_AJTT01000004	65172 to 65365	CCCGTGGCTTGACCACAATATTTAGAGAAATAACTTGTAATAGTCAAATTTACTAAATCCTGCAATCAAATAGCGGGAGATGACAGGGGCTGAATTGATCCACACAGTCAATGCCTGCTCGCAATGACGAAAAATACACTATAAACTAAGCTTCTTTAGCTTCCTTTTTACTTAGTCTAGCTTTGCGGTTTTTTG
LH08G:00314:02276	289	96.181	NZ_AJTT01000004	58769 to 59047	TAACTCATTCAATATTATTATTAAGCAAGGCAATGGGTTATTTGCTTTATGAGGATAAGCTACATGCCCACTTAAGCCTTCTATATTTAATTTAAAGTTAACACTTCCTCTTCTGCCAATCTTAATTGCATCGCCTATTTCTTTTTTCACAAGTAGGTTCACCGACAATAGCAAAATTTTATCTTATATCCTTGATCATAAATATATTGCAGCATTTCTTTATACCATGCTTTTGTCTTTTTCCCTCTTTCACATCTAGTAAGTAAAGAAACTAATGGAACCCTTTTAA
LH08G:00420:00401	207	100	NZ_AJTT01000004	65318 to 65524	TGCTTGCTTTAGATAATAGTGAGCGTGTTGTTTTAAAAAAAGATCGTATAGAATATTGGCTTGGCACCGGTGCTAAACCAACTGAGCGAGTCGCAAAATTTATTGAGCAAGCGGGTGTTACTCTTCCTGAGAAAGTTAAAAAGGAAATGGAAGTGAAAGCAAAAAACCGCAAAGCTAGACTAAGTAAAAAGGAAGCTAAAGAAGCTT
LH08G:00456:01603	256	99.609	NZ_AJTT01000004	88760 to 89014	TAAATTTTTTAATAAGTTTTGTTGTTTTTCTTGCAATAATTCCCTTACCTCGGCAAATGCTTTGTCCGTATCTGCGTAGCTTGCAGCCATATTCCCATTATTATCAGCACGAATAATCATCTCAGGTGGCAGCAGCTTAATAAACTCTAGAGAAACTGCTATTATGCCTTTTTTGGATGCAAGCATTAACAATGTCCTTCCGAATTTTATCAATAGAGTTAATATTTTCCTCAAACATTCTTGGAATAAGCTCTAT
LH08G:00479:02448	249	100	NZ_AJTT01000004	154629 to 154877	AAAAATGACAATTATCTTTCGGAACTAAGCAAGGTAATTTGGCCTATAGAAAGATATGAAAATAAGAAGTTTCTTCCTATGGCGTTTATGATGTTCTGTATTTTATTAAACTACTCAACGCTTCGTTCAATTAAAGACGGGTTTGTAGTAACAGATATAGGAGCAGAAGCAATAAGTTTCTTAAAAACATATATAGTACTACCTTCTGCCATAATCGCTATGGTAATTTATGTTAAGCTCTGCGATATT
LH08G:00595:00466	205	96.098	NZ_AJTT01000004	105735 to 105931	GAAGTTACATTAGAAATTAAACAAAAAAATACAAAAGAATGTTTTGAATAAAATATATATTAATGCTGCTAAAGATAAGTGGAATAGTAAGTTCTCAATCTTAGGAAATTGGGGGGTGCAAGGCTATTTAGGTGATAGATATTTTATAGCAAAAACAACTAGGAATTTTTATCTGACCTTAAAAAAATATAGAAAATAGCTAAAA

Read name	Sequence length	% ID	GenBank accession no.	nt coordinates	Sequence
LH08G:00622:00271	266	99.248	NZ_AJTT01000004	60676 to 60940	ACCCTTAAAACTGATCAGCAGTTAAAGTTTGATGTAATTGTTACTAATTGCCATTTTCTCAAACTATCACTAAAAAAACTAGCAAAAACGGCAAAACTATAACAGAAAATCATATAACTTCTCTTTTTAAATAACGGCATTGCAAAAAATAACGGTGATGCTGTTTGTGTTTTGCATTGCCTGCAAAATTTAAAAGAGGGTGGTAGAATGGCATTGGTAGTACCTGAAGGGTTCTTATTCAGAAAAGACACCGCTGCTGTTCGTCA
LH08G:00812:01794	217	100	NZ_AJTT01000004	26095 to 26311	CATTACTTTTGATAATGTCTATAGCATCAAGACTTATATCGGTTGCAATTACATTAGCATTTGGTAACTCACATAATAGACTGATAGCAATGCAGCCGCTACCTGTGCCAAGTTCTAGGATATTTAAGAATTTGTCATTGCAGTTTTCGCTTATGTCATTCCCGCGAAAGCAGGGATCCGGTCGCTTTTTTTCTGGATTCCCGCTTTCGCGGGAATG
LH08G:00834:01577	228	99.123	NZ_AJTT01000004	51094 to 51319	GAGAAGGGATAATCATATCATTTAGTTTATCTAATTTTTGCTCTTCTGTTAGCTCTTTTTTTACCGGTTTCTAAAAAATTTTTTCAAATTACAAGCATCTTGTAGATAAAGTTTAACAACATCAATATTATAATCTTATTATTGACAAGTTCTTTGATCTTCTTAATGTCTTCATTGTAAATAGCATTAAACAAATTGATTTCGTCATATTTAGATTTATACACAAAA
LH08G:01093:03061	337	99.703	NZ_AJTT01000004	123035 to 123370	CATGTAGTCCCGGTACTATTTGAGTATCGTTTGATAATCTATTAGGAGCGGTTAATTTTATGCCTGCAATCCCTGTAGTTAAGTTACCGGACTTACCAGCGACATACATATTATTCACACCTGTACCGGTTTGCTGCAGTATAAGTATATCCATTAGGAACAAATCCATCTCCATTACTCATCCTCAAAATTGATAGGGCGGCTACATCATTCGCCGTGTGAACCCTATTAATATTAATCACAATATTTGTACCGAATGCCGTAGGGAGATTTTGAAAAAACTATACCGCTTTCTCCTAAAGCATTTATAGTAATATTACCTTCAAGTACTACATCA
LH08G:01130:00477	302	98.675	NZ_AJTT01000004	133188 to 133486	GATATTAGTAATTAAAAATTTATTTACTGATATCCTCTAAGAAGAAATATAATTTTTTTAATATTGACACTTCTTAATTTAGTAGTATGATATGTAATTACCATGAAACATGGCAATTACGTATATAAAAAAGAGGTAAGTTTATGAGCAATAGCAATTATGAAAACATATACAAAAGCATTTAATTGTTTTTCAGAAGCAACCAATTCTGCCTATTCAGCCGTAAATACTTTTTTAAAACTACCTAATGTATTTAATAAATATCCTACTATTATTTCTTTAATTTTTTTCTAAAACAGCAAC
LH08G:01200:03346	198	97.475	NZ_AJTT01000004	138841 to 139033	TACGCTTGTGTACGCTGCGGTGCTGCGTTCCGTGTCTCCTTTAAATTCCTCTCTATAAACTGGTTTGGAAAAATTGTCTAATATAATCTGCATCCTTTAGCTAATTTTTGCCTAATAACATTTCGTCTATATTTATCAGATGAATTTGAACTCATCTTCAAACCTATTTTATATTTATGACTGACTAAATATTCTACT
LH08G:01395:01645	286	97.909	NZ_AJTT01000004	88415 to 88696	TACTTTTTTCCAGACAACTCAACATCTATCCCCCATTTTTCATTTTCTAAGTCGGAGATAATACACATATTGCTTCATTTGGTAAGCAGTGATAAATGTAAAAAAGGGGGGGTAGAATCCGATTGAATTTCCGGTTGTATATACTCCTGCAATGGCAAATGGGAATAATTTCATAAAATTATTCATCGTTATTATAATAGATTCAGGATTATTATTTTGTTCTTGTAAATAAGATGCTAATAGTTCCTTATTTACTTTCACTTTTTTTTATAGAATTTTTGATAGCA
LH08G:01446:03084	391	99.225	NZ_AJTT01000004	77026 to 77411	AATATAAACATGGGATAGTATTATACACGATTGCCGATCAAGAACTCCGAAAAACTTTAACGAAATTTTGCTATGAATTAAAAATTCCATGTATTTCTGTAATAGGTAAAATTATTAAAGAAATGTCTGTTTTTTTCAGGTATTGAAATAGAAAAAGAACAGAATTACAATTATAAATTTGATAAAACTTATTTTGATACACTTAATGCCATAGATTATGCTATAAGACATGACGACGGACAAATGCTTAATGAATTATCAGAGGCTGATATAATATTAATAGGTCCTTCTAGAACTTCTAAGACACCGACTTCCGTATTTCTAGCGTATAACGGTTTAAAAGCTGCTAATATTCCTTATGTTTATAATTGCCCCCTTTCTGATTTTTAAG
LH08G:01533:00837	279	99.283	NZ_AJTT01000004	144101 to 144377	ATTCACAATCAGCATCTCGGTACTTAATCCATAAATTTGGGGATTTTTTAAGTAAATTAACTTGTTCTCGTTTGTCTGAAATATGTTTTAATATTTCTTGGTATATTTGATTTAGTTTCTTATCAACTTTCTTATATTCTGCAGCGACACAATAATTCATGTCACCGT

Read name	Sequence length	% ID	GenBank accession no.	nt coordinates	Sequence
LH08G:01668:00801	198	98.99	NZ_AJTT01000004	76552 to 76747	GTGTCATGGTATTATTACAATCAACAGAAAATAATTGCTTTTTTTCATTATTTTATCATTTTTTAAAAATAATTAATTAAGAATCACACACCGTGACGTTCTGCAATTAAATTTAAATAAATAGTTTGTGGATAAAATTGTGAATAAATATCATTTTTCTTTGAAAAAATAGTCTTTTCTAAGCTAGAAATGAATTTTACACTTAATTATCCACTTGTATAAATTCATTACAAACTTATGTGAATAATAATTAAAAATACCTATATTTTTAAGAAAATTTTAATGAAAATTTTGTTTGATAATCTCTGT
LH08G:01741:01988	263	100	NZ_AJTT01000004	9014 to 9276	ATCCACGTTCTACGGTTGGAACTATAACGGAAATTTACGATTATCTTAGGTTATTATATGCAAGGGTGGGTATTCCTTACTCTCCGGCAACCGGTCTTCCGATACATAGCCAAACAGTTTCTGAGATGGTAGATATAATTAACGAACTACCTAAGGGTACGAAAATATATTTACTCGCTCCTATTGTTAGAGGACATAAGGGTGAGTTCAAGCGTGAAATTATGAATTTGAAAAAGCAAGGCTTTCAGAAATTAATAGTTAAC
LH08G:01837:01796	230	98.696	NZ_AJTT01000004	124826 to 125052	GTTACTTCTTTCTTAACCGGCTGTAGCTTTTGTATTTGCTTTTTTTAAATTATCGAAAATTGATTTTTATTTTTTTGATCTGAAGCGGAATTTCTGTATAAATTTTGAAAAAGCTTGGTAGTTTCAGGGATTAAATGCGGGCTTGTTAGCTCTTCTTGACTAACCATTAAGCTTATATTAAGAGCAGGTTCAGTAAACCTTGCAAAATTGCAGCAATCATCGGTATCTAT
NB501781:2:HGMVWAFXX:4:21410:9055:4052_1:N:0:TAGGCATG+ATAGAGAG	151	100	NZ_AJTT01000004	71988 to 72138	GAACAAAGATTTACCGGTGTTGCTTTCAGCTACAATTTTGCAATAGCGATATTCGGCGGAACTTCACCTATTATTTCTCGTTGGCTTGTAGAGCGCACAGGCTTATTTTATGCTCCGGCTTTTTATATTATGATTATTGCTGTCGTATTTT
700666F:214:CA2UUANXX:8:1310:15115:58891_1:N:0:TAGGCATG+CTCTCTAT_(reversed)	125	100	NZ_AJTT01000005	47603 to 47727	CCGTTTCGGTTTAGCCTTAGATGACAAAGGCCATAGTCTTTTACCGTTACCTCCTGCCATAATCACCGGTTTTATTAGACTCCTTGCATAACCTCCTTTAAAGGTAATTTGTATACCTTTGATAC
700666F:214:CA2UUANXX:8:2311:18340:31562_1:N:0:TAGGCATG+CTATCTAT_(reversed)	125	100	NZ_AJTT01000005	78091 to 78215	ATTAATTAAACCGATTGCGATGCAGGAAGGGTTAAAATTCTCTATACGTGAAGGTGGTAGAACAGTGGGTGCCGGCGTAGTAACTAAAATAAATAATTAATTGATTTTATAAATACCTATTATTT
LH08G:00199:02588*	272	100	NZ_AJTT01000005	3892 to 4163	GAAGATTATTATATGCTGCAGTATTAGTTGCATTTACAAATGTTGTAACGTTCTGACCTACACCCGGTGCAACTCCAAACGGACTATTTGCGATATCATTAGTAACTACGTTTTCTGCATTGTTAGTACGTGTTATTACATAATCTTGGTTAGCAGCACGTATTAAACCGTAATTTACGAAACGATTACTTCCGGTTACAGCAAAGTTGGGACCTCCTAAAGTACCGTTAAATCTAGCACCACCTTGGATTAAAGTATAAGTTTGTGTACCA
LH08G:00350:00627	165	100	NZ_AJTT01000005	95517 to 95681	CAAATGGAGCGAATAGAATGGCAAGACCTATAAAAGAAGAAAATAAATATGCCACACTCACTAGAATAGTCGGTAATAATGTAGCACAAATAATTTCATCAATAAATATAGGATTACAATCATTAAAAGGTTCTAAAGATTCGGTCAATCAGATCCAAGATAGGA
LH08G:00551:02828	401	99.499	NZ_AJTT01000005	24341 to 24739	GGATTATAGAAGTAAAATCGATAAAATCTTTTTTATCAGCAATATTAGGTTGAAGTAACATATTATACGCAGTAGAATTAATGGGTCCTATTTTTAGACAATCCCCTAATTTAGCTGCTATTTTAATGAATATTAAAGGAATTTTTATTAGTCTTCCATGACTCCAAGCCATCTTCTGAATCCAAGCAAGATATCCTTCATGTTCACTATGTCTGGTCCAACTATTTTTAATAATGTGTGGATTTTTCCTTCCCTTTCTATACAGTGAATCATTACTTTCGTTAAATCATCAATATGAATAGGTTGAAATTGTTGTAAACCATCACCTATTAAAGGAATAAAATACGGTAATGTTGCAAGAGCCTAAATAGCGAGGTACCACCATAACAACCGCCTGCTGT
LH08G:00753:00799	191	99.476	NZ_AJTT01000005	101236 to 101426	ACAAATTGCGCATATTGATCAGAGGATAATTCTACTAATAAGGCATAATAGACAATAATATAATCTTAATACCATAGCCGCTTAAATTTAAAAAATACACTATAATAGCAATTATAAATTGAGTAAGGAGCTGGGTTAAAAAATTTTTAATTGAAGTAGTGTATGGCCTATTGTAGTAATGATTTTATAAC

Read name	Sequence length	% ID	GenBank accession no.	nt coordinates	Sequence
LH08G:01200:02029	304	100	NZ_AJTT01000005	30588 to 30891	GATATAGTCATTGTGAGTTACTGCAGGTGTTGTTGCATGGCTCGTTTTATGTTATGCCTGCGCAGGTCTTGTTGTGTGGACCGAAAAACCCGTTCGGTGTTATGCCGTGGCTTGACCACGATATCCAAAAACACAACTTCAAAATACTAAAATTTTAATGTTTTAAACTGGATCCCGCGATCAAGTCGCGGGATGACAGTTAGGAAACTGGTCCACGCAACAAAGCATTCCCGCTTTTGTGGGAATGACATCGGAAGGCTAAACTATAAAACAACTTTTTCTAGGCAATGCTCAATTTCAATAA
LH08G:01287:00555	110	97.273	NZ_AJTT01000005	134197 to 134303	CAACTGAAAGCTTTTTATTCTCGTATGATGATTCCTATTATTAGACTTCTTGCATAACCTATCTTAGAAAGAGGGACTTGAAAGAAAAACGGTAAGCACTTGCCACCGCA
LH08G:01331:00575	339	98.525	NZ_AJTT01000005	260 to 596	ACTAAATTTTTAACTTAAATATAGGACAAAAATTATGGCTCAAAAACCAAATTTTCTAAAAAAATTAATTTCCGCAGGGTTGGTAACTGCTTCTACAGCTACCATAGTAGCCAGTTTTGCAGGTTCAGCTATGGGTGCTGCTATACAGCAGAATAGAATAACAAACGGAGCTGCTACAACTGTTGATGGTGTGGGATTTGACCAAAATGCCGCTCCTGCAGATGTTGCAGTTGCTCTAAATGCAGTTATTACTGCTAATGCTAATAATGGTATTACTTTAAAATCTCCAGCCGGTAGTTTTAACGGTTTTGCTTTTAAATACTGCAAACAATTTAGTAG
LH08G:01354:00607	218	100	NZ_AJTT01000005	103977 to 104194	ACCGTATAAAACTTTATCGCCTACTTTTAGCTCTAAAGGATGAATTTCGCCTTTTTTATTACGAATACCATTACCTACGGCTACTATTTCACCTTGCATCGGCTTTTCTTTTACAGTATCCGGAATAATAATTCCACCTTTAGTTTTTTCTTCTTGTTCGATAGGCTTTATTGCAATTCTATCATGTAATGGTTTAAAAGACATTTTGACCTCCAATT
LH08G:01628:02693	228	100	NZ_AJTT01000005	65377 to 65604	TATTTGAACTTAACACACGCTTATAATAACTAAAACTTAAATTTTACTTTAAGCCTTACTTAAATTAAGAGCCGATAAAAATTCATTATGCAAAACTACCTTACCTTGCGGCATTTGTCAATAAACATGTCAATAACATGCAAGAGAAAGTATTACTTCCTGAAACTAATCTATCATATTATTACTATTTTGTCACTATTTAAGTTAATTATCTAATAAATATAATGA
LH08G:01989:01385	211	100	NZ_AJTT01000005	69712 to 69922	AAATCTTCCTCTACAGCTTCAACTGCTTGCACTTCCGGTACAAAATGTTTAAGCATGGACTCAATGCCGTTTTTTAATGTGATGGTGGAACTAGGACATCCAAGGCATGCACCACGAAGTGCTAATTTAACTACACCGTTTTCAAAGCCTTTATAGATTATGTCGCCGCCGTCTTGTGCAACAGATGGACGCACCCTAGTTTCAATAATCT
LH08G:00023:01565	289	100	NZ_AJTT01000006	54341 to 54629	CAAGTCCGTCAATTTCGCTTCCTTGAAGCCAAATATCAAGTATAACTGCAGAGACCGGTTTTTCGGAAAGTATTTTAAGGGCTTGAGTACTATTAGCGGCAACCTTAGGGTTAAAACCTTCATCTTTCAAATTTGCAGCAATGAGATTTCGTATACTCTCTTCATCGTCTACTATTAAAACATCTAGTTGTGACATTACAATTTACCTTAAAAATATTAATACTAGGTTCTTACGTACTATTGCTAATTGATTTAATATTTTACGATATTTTTTAGCGAAAATTAATAA
LH08G:00130:00422	198	98.99	NZ_AJTT01000006	27847 to 28042	TACTACATTATGATCAAATTTTATAGAAAAAACACGATGTAATTCTACGCTTAAATTCTCTATATCCCAATCTTCTCTATAAGAGCCTACCGGCATGAAAGTTAATACTATCTTTTTGGCGAGTTCCTCAGTAGTACTATTTAAGAAAACGTGACTATCTTTAGATTTAATAATTTCAGTTACGTTGTCTCGTATATT
LH08G:00515:01357	273	99.632	NZ_AJTT01000006	74345 to 74615	AGTTCTTGTGAAGTTTAAAGGTGTTAACCATAAAGTAGTCGAGATATTGATTGATAGCTTAAACAGCGAAAAAATATCTGTGGAAGATTGTACTAAAGCAAGTAGGACTATCTCCGCTATTCTAGATGTTGAAGATTTAATAGAAGCTGCATATTCTTTAGAGGTAGCATCAAGCGGTCTTTGAACGTCCGTTAGTAAAGTTTGAAAATTATAATAGATTTTTAGAAAGAGAAGTTAAAATCAAACTTAAAGAATTATTGAACGGCAAAACTT
LH08G:00539:02467	256	99.219	NZ_AJTT01000006	88920 to 89173	ATTATTTTAGCTAATCTATGCATTTTTAAATATAGTAATTTTCTAATAGACCTCTTCCCAAGCGTCGCTTCTAGAGATAATTTATACGTCGATTCGGTACTCGCATCTTCACGTATTTCTATATATGCTGTGGTGCTGTGTCCCGTGTCTCCGTTAAATTCCTCTCTATAAGTTTGGTTTGGGAAGAGGTTCTAATAAGAATATATAAAAATACCCTTAAAAAGCAATATTAATAATTTTATATAAATTATTAATA

Read name	Sequence length	% ID	GenBank accession no.	nt coordinates	Sequence
LH08G:00988:02633	327	100	NZ_AJTT01000006	31603 to 31929	ACTCCCCTTGATACCTACATACCAAATCTTACAGAGTCAAGATTTTGTAAATTTTTCTTTAAGTCTACCAGCGATGTGCCTGCTACTAGCTTTCCTATATCGCCTGCTATCTGCATAGTCTGAGTGCCTAGCTGGTATAAATTTAATGCCTGATCTAGAACATCAAGCCATTGCGTACCATTTTTATGTATTAAGTGATTGGCAAGCTTTGCAGTGTAGCTTAAATCTTCTTGTATGCCAATATGTATTGTATGCCAGCTTGCATTGTAGTAGATAGATAGAGGATTTAGCTGTCATTACCTATTATATCGACTTCGTCCTCTAAAT
LH08G:01328:01305	274	99.635	NZ_AJTT01000006	98551 to 98823	TCTAGAAAAATTTCAGAAAGAAGTTGGAACGGATGATATTGTTAGTGTTACTCCAAGTCAATTAGGTCTAGCTAAAAAAGCTAAACAAAAAGACCAAGATTTATTAAAATTAGATGAAGAAGATCTAAAACATATAAAGAATCTAAAAGAAAAGGTTATGAAGCCTACTCAAACTACTGCAGTTAGTGTTGTTAATAAAAAAGTCTACACCGGCTAAAATGAGATAATAAATGAACCAAGCAACAAGAAGCTTACAACTAAGTAATTAATCAGT
LH08G:01472:00458	205	100	NZ_AJTT01000006	99316 to 99520	AGCATAGATAATATAGCAATTATCTTTATTAAATTATAATTAATAGGATAGTAATTACCTATAATTAATTTGCTGATATTGAATAAAACAATAACCATACCTATTTTTGAGGCAGCATTGAAATAAGTAACCGAAGTAATCGGTGATCCTTCATATACGTCAGGGACCCAAAAAATGGAGTGGAACACTTGAGAGTTTAAAGAAAA
LH08G:01636:03017	282	100	NZ_AJTT01000006	62415 to 62696	GTGGATTTAGATATTGGAACAATTTTAATGACGATTCCGATCCATTTATTTCCGTTTATGCTATGCATTTCTTAAGCGAGGGCGCAACTAGATATTTAGCCGTTCCAAGTGATACATTTAATCAAGGTATTTACTATTTAGAGAATATGGCTAACAGATCAATAAACTCCTTAGATGAAGCAAGAGAAAAGGCATATGCGGTTTATATCTTGACGCAAAACAGCGTTATTACTACAAGCTATATTGCAAATATTTTAAAATATTTAGACGAGTACCATAAAA
LH08G:01751:03116	201	97.015	NZ_AJTT01000006	96058 to 96252	AGCAAAATTTCTTTTTTGTAATGCAATATTTTTACTATAAGATAGTTAGGTTATATATTAGAATCCAAGTTATTAACTAAAATGAGATATGAATTTTATGTACCCCCATATTAGACTTAGAAAGAAATAGAAAAAGCTTTTTTGGCTTAGAGAGTTTAGTATCAGAAAGTAATTTATCGGTTTAGTGATTTAGTGTTGCCT
700666F:214:CA2UUANXX:8:1109:9570:7533_1:N:0:TAGGCATG+CTCTCTAT	125	100	NZ_AJTT01000007	42739 to 42863	TTGTCACGCAGCCTATGGCTGCTCGCAATGACGATTTGGTATCCATGCAAAGCCTTAAGCAGGAATGATATCGATTGTTCTCTCAATTCTAATACAAGCTTTTTCTAGCTCTTCCATAGAAGTAG
LH08G:00493:02627	249	99.197	NZ_AJTT01000007	30847 to 31093	TGAAGAGTTGAAGTCGCCTGAACTAATATTGCTACTTTTCCACTAGTATCATCAGATACCTGTTGTCTCTGTGTCACCTCATCTTGTTCTGTAATTGCTTCAGTTTTGAATGACACATCAATAGCTTCATCTTCGCTTTTCTAAAACTATATCTTTTTTTAATTCTTCGTCAGTGGTGCTTATTGCTTTCTTCTAATTCTTCTTCATCAGTTGCATACCCTTTATCGTCTTCATCACTAGATCTTAATG
LH08G:00605:00996	178	98.876	NZ_AJTT01000007	59163 to 59338	GACAGACAGGCATTTATAATGTTTTAACTGCTGCATAAAATATAGGTAACCATTTATCCGGATCATTGCCGTGTTTCTCTCTAATTTGATCGAGCAATATTACAGTCTCAACTCTACCGGATAAAAACACTGATTTATGTTATCCATATCATCTAAGCTAACTTTTGCCACTACAGCA
LH08G:00669:00823	208	99.519	NZ_AJTT01000007	1664 to 1870	AGAGTAGGCACTCCAAGACGTGCAGAAGCAGCGGCTGCTTCTACGCCTGCATGTCCACCGCCTATAACTATTACGTCATATTTTAGCATTAATTATTCTATAATTTCAGATTTATTAAAGAAAAATTCTATTTCTCTTTTTCGCACTATTTTCACTATCTGAACCATGAATACTATTAGCCTCAATTGATTCGCCTAAATCTTTTCTA
LH08G:00671:00991	332	99.699	NZ_AJTT01000007	15458 to 15788	TACCGAATCACGTATGACGGAAATATGTACGTTATTAATGGAGGACATCGATAAAGATACGGTAGATTTTCGCCCTACTTATGATGGTTCCGATTTAGAACCGGTAATAATGCCGGCAAGTTTTCCGAATTTACTAGCTAATGGTTCTGAAGGGATAGCAGTCGGTATGGCAACCAATATACCGCCGCATAATTTACATGAGCTTTGTGATGCTCTAATACATTTAATCAACCATCCGAAAGCTGAAATTAACGATATAATGAACTTTTGTCAAAGGTCCTGATTTCCCGACCGGCGGCATAATTATTGATAAAGCTGAAGTTATTAATGCT

Read name	Sequence length	% ID	GenBank accession no.	nt coordinates	Sequence
LH08G:00721:01865	328	99.695	NZ_AJTT01000007	52489 to 52815	CCTAATAAATATTGCTGAATAATATGTTTTTGTTGGATTTCATTTAAAGAGCGATTAGGTATATTTTTATTTTTAGTGAAATCAAAAGCTTTATAAAGTACAGGAGTATTACTTCCTATACTTAAATGTACTTGTCTAGCAGCGGTTGTTTTATCCTCTCTATCCGGAACAGTTACCTGTTATTGGATCAAATATAATTCCTAATGGGGCTCCCTTGTCTTCTATATCTGCAGATTTTATATTTACGTTAAAGTGGTTAGTTAATTCTTGTGTTACTTTACGAATATTACCATCTGATACCGCAATTTGATACTGCAGATCTATCACG
LH08G:00727:00743	311	99.678	NZ_AJTT01000007	49313 to 49622	GCGAGTATTATAGGTACTATCGGGTATGAGGTATTAACTACCCTTGGTAGTAGATACAAAAGAAAGTATATCGGGTAGATATCTGTGTCATACCATGGCTTGACCACGGTATCCAGAAAAAAATTAAAAAAGACTGGATCCCGCGATCAAGTCGCGGGATGACATTAGATGTTACTGGATTCCTGCTTGCGCAGAAATGACATAGCGTACTTAAATTAAATAGAAAAAATACAAATGTTATTCAATATAGCTAATTCGGTCGGTAAACGTACTGTAAAGTTTGCACAAAGTGTAGGTAGTTTTTCTCTATT
LH08G:00910:00305	279	99.642	NZ_AJTT01000007	40345 to 40622	TAAAGATACATTACGGTAGAGACGGAAAATTAAATAAGTTATCGTTGCCAAACCCACCTATAATTTTTCATAACCAATGGTATCCGGCCTTAACCGTTTGTAAAGGTGAATTATGTTCTTTGCCTATAAGTAGTAGGTATTATAGGTATTTGAATAAGAAAATACTAGAAAATAACGGAAGTATAGAAATCAGTCACGTAGACCCTGAATTTACTATAGAGCTTTTAGGAGAATAAGAGTTTTTAGTTAAATTTTATCAAAGAAACTTCGTTAATTACT
LH08G:00990:02358	303	99.67	NZ_AJTT01000007	50872 to 51173	ACTATTACGCATTTCATCTTTACTACCGTACCATTTAATTTTTCCTTGATAAATCATAGCTACTTCTTTAGCTATTTTTGCGGCACTAATCATATCATGAGTTATAGTAATCGTAGTTGCCTCTAACTCTTCTTGTATTTTTATAATTAATTCGTTGATAACATTTGCCATGATAGGATCAAGCCCTGTGGTCGGTTCATCAAGCAATAAAATCGACGGTGTACTACAAATAGCCCTAGCAAGGGCTACTCTTTTTTTGCATTCCTCCCGATAATTCGGCAGGGTAAAGCTCGAGTATTCTAG
LH08G:01166:02332	83	98.75	NZ_AJTT01000007	47634 to 47712	AAAAATTTCTCTACTTGTATCTGAGTGTAAATCAATAGTAATTACATGGTTTACTCCGAGCTTCTCTAAAAAATCTGCTACTT
LH08G:01538:00955	329	98.784	NZ_AJTT01000007	50655 to 50979	GTTCATCAAGCAATAAAATCGACGGTGTACTACAAATAGCCCTAGCAAGGGCTACTCTTTTTTTGCATTCCTCCCGATAATTCGGCAGGGTAAAGCTCGAGTATTCTAGGAGATAAACCAACGGCATTAAGCTTTGCACCGGCAAGGTCGTTTTTTTTCTTTCTTAGATAATTTTTTTAATCTCGAAAGTAATATTATCACGTATATTTAAGGAGTCAAATAATGCTCCGCCTTGGAATAAAAAAACCTATACCATCCATAATCTCAAATTTTTTGTTTACTTGAGATATCTTGGATTTCTACATTATCAATAAAAATTTTACCTTTATC
LH08G:01900:01532	178	98.876	NZ_AJTT01000007	61066 to 61241	TATACAAGAAATTATTTTGAGAGCTGGGTTAAACTAATGATCTCGTTCTTGCTGCAACCTATGGTGGTAGTTACTTTTTATGATCACGATGTTTTCGGTATATGATTATGGTTTCTACGGTAAATGTCAATATAAGAGCAAGTTAATTCACAATAGTATAGAAGACAAAAAATTCAAGG
LH08G:02043:01078	257	100	NZ_AJTT01000007	60308 to 60564	GGCAAATGATTTCTAGATTACTAATTAGTAAAGATGTTTGTAGTTTTGATAATGTAAGCCAAGTGGTTAATTTGGTTTCAAGGCAAGATAGTGCATTCTTTCAGTTTCAAGTAGGAATGTATAAAATAGTTACGGCTTTTCTAACTTTATATGTTATGTTTTTTGGCTTTAAACTATTACTTGCAGGTGAAGTGCCGCCAAAAAGTGAGTATATAAATTTTATATTGAAAATGATATTCGTAACTTATTTTTCAATA
700666F:214:CA2UUANXX:8:1108:8596:42754_1:N:0:TAGGCATG+CTCTCTAT	125	100	NZ_AJTT01000008	25142 to 25266	GTAAATATCCGCATCTACTATCCCGACTCGGTAATTTGCTAGACTTAACTGTTGAGCGATAAGAGCAGATATTGTAGATTTTCCGACTCCGCCTTTACCTGACGCTACTAAGATAATTTTTTTTA
LH08G:00129:01101	116	100	NZ_AJTT01000008	20602 to 20717	ATTTAGACGTCGATCCAGTACTCGCATCCTCACGTACTGCGGCGGCAAGTGCTTCCGTGTCTCCTTTAAATTCCTCTCTATAAGCGAATTTGGCAAGATGTCTATTAGTCATTTTT

Read name	Sequence length	% ID	GenBank accession no.	nt coordinates	Sequence
LH08G:00238:00771	252	100	NZ_AJTT01000008	17949 to 18200	CTTGTAAGGATCGTGATATATTAAGGTTTGAGCCGCACAAATTAATAGAAGGTT GCTTGCTTGCAAGCTTTGCTATAGGAGCGAATAATTGTTATATCTATATTAGAGG TGAGTTTTATAATGAAGCCTCTAATATTCAGCGTGCCTTAGATGAAGCCTATAAA GAAGGGTTAATAGGAAAGAATTCTTGCGGTTCGGGTTTTGATTGTAATATTTATT TACATCGTGGGGCGGGTGCTTATATTTGTGGTG
LH08G:01472:03357	329	100	NZ_AJTT01000008	39709 to 40037	AGCCGTTTAACGGTAGTATAGTAATAATAAAAAATTTTGTTATTTTAAGCATTTGT ATCAGTTTAAATATATTAAACTGTATTATAAAAGGAGAATTAAAAGGTGTTAAGAT CAGAAAAACCGGTAGCAGTAGAGGATATTGTAAATATTTATAAAGAATCGCCCT CTATAATTATTACTCATTATCACGGGTTAACCGTTAGTCAAGTGAGTTCGCTTAG AGAATCACTTAAATCTAAAGAAGCAGGTTTTAAAGTAGTTAAAAATACTTTAGCA AAAATAGCTGCAAATCAAACAGGGCTTAATAGTATTGCTAATTTATTTGCGGG
LH08G:00343:01258	271	100	NZ_AJTT01000009	20861 to 21131	AGTGAAATATTAGAACTTGAAGCGGAATCCCGCACGGAATTCGGTACGGGAGC AGCAAGAGCATTAAGAAGAGCAGGGCGTGTTCCGGCTATTATTTATGGAGCTG GTAAAACACCTGTTAGTATTTCTTTGGAAGAAAAGGAAATAACTAAATATTATAG AAAGCCGGCTTTTATCTCTCAGTTAATTAATTTAACAATTGATAAGAAAAAATATA AAGTATTGCCGAAGGCTGTAGAATTACATCCTGTTACGGATATAGTACGCCATG
700666F:214:CA2UUANXX:8:1215:13592:16288_1:N:0:TAGGCATG+CTCTCTAT	125	99.2	NZ_AJTT01000010	7225 to 7349	CCCTAAAATATCTCAAAGCGAAATAGCAGAATTGCCGCCTATTTCGTTATTACGT GATCCTGAAAAACATCATGTAAAAGGAGCTTCTTCCTCAGAGCTTAAACAAAAG GTTGAAGAGTTATTAA

*Gene name: *ompB*.
